# Seasonal metabolic dynamics of microeukaryotic plankton: a year-long metatranscriptomic study in a temperate sea

**DOI:** 10.1128/mbio.00383-24

**Published:** 2024-07-09

**Authors:** Michiel Perneel, Rune Lagaisse, Jonas Mortelmans, Steven Maere, Pascal I. Hablützel

**Affiliations:** 1 Flanders Marine Institute (VLIZ), Ostend, Belgium; 2 Department of Plant Biotechnology and Bioinformatics, Ghent University, Ghent, Belgium; 3 Center for Plant Systems Biology, VIB, Ghent, Belgium; 4 Biology Department, Vrije Universiteit Brussel, Brussels, Belgium; The University of Tennessee Knoxville, Knoxville, Tennessee, USA; The University of Tennessee Knoxville, Knoxville, Tennessee, USA

**Keywords:** microeukaryotes, metatranscriptomics, seasonal dynamics, marine plankton, ecosystem monitoring

## Abstract

Seasonal fluctuations profoundly affect marine microeukaryotic plankton composition and metabolism, but accurately tracking these changes has been a long-standing challenge. In this study, we present a year-long metatranscriptomic data set from the Southern Bight of the North Sea, shedding light on the seasonal dynamics in temperate plankton ecosystems. We observe distinct shifts in active plankton species and their metabolic processes in response to seasonal changes. We characterized the metabolic signatures of different seasonal phases in detail, thereby revealing the metabolic versatility of dinoflagellates, the heterotrophic dietary strategy of *Phaeocystis* during its late-stage blooms, and stark variations in summer and fall diatom abundance and metabolic activity across nearby sampling stations. Our data illuminate the varied contributions of microeukaryotic taxa to biomass production and nutrient cycling at different times of the year and allow delineation of their ecological niches.

**IMPORTANCE:**

Ecosystem composition and metabolic functions of temperate marine microeukaryote plankton are strongly influenced by seasonal dynamics. Although monitoring of species composition of microeukaryotes has expanded recently, few methods also contain seasonally resolved information on ecosystem functioning. We generated a year-long spatially resolved metatranscriptomic data set to assess seasonal dynamics of microeukaryote species and their associated metabolic functions in the Southern Bight of the North Sea. Our study underscores the potential of metatranscriptomics as a powerful tool for advancing our understanding of marine ecosystem functionality and resilience in response to environmental changes, emphasizing its potential in continuous marine ecosystem monitoring to enhance our ecological understanding of the ocean's eukaryotic microbiome.

## INTRODUCTION

Marine microeukaryotic plankton play a pivotal role in primary production, global oceanic biogeochemical cycles, ecosystem stability, and climate regulation (1–4). They exhibit pronounced spatiotemporal dynamics with recurring seasonal phenological patterns (5). Such phenological rhythms, like the yearly blooms of both phototrophic microalgae and heterotrophic grazers, are particularly sensitive to disruption by anthropogenically altered environmental drivers (6–9). In the face of current climate change, understanding the patterns and drivers of seasonal microeukaryote ecosystem dynamics, i.e., cyclic variations in species composition, biomass, and metabolic activity, is paramount for deciphering marine ecosystem functionality and resilience (10).

Recent advances and maturation of methodological approaches in the marine sciences, including plankton abundance time series (11, 12), large-scale -omics surveys (13–16), and expansion of genetic reference databases (17–21), have been propelling a deeper understanding of marine ecosystems. However, many studies only track natural microeukaryotic plankton assemblages over limited periods of time (22) or only at a few dates of the seasonal cycle (23), whereas complete seasonal trajectories are needed to fully understand the temporal dynamics of plankton assemblages and the associated metabolic functions. Metatranscriptomic profiling provides an underused opportunity for a more comprehensive characterization of coastal microeukaryote plankton ecosystems (24–27). The systematic capture of microeukaryotic plankton gene expression profiles over time from fixed locations generates ecosystem snapshots that facilitate the reconstruction of the turnover and relationships of active species and their metabolic responses to environmental fluctuations.

In this study, we aim to capture the phenological patterns and metabolic functions that follow seasonal turnover in temperate microeukaryotic plankton assemblages of the coastal Southern Bight of the North Sea. This shallow marginal sea is characterized by a complex subtidal sand bank system and high nutrient input from the Rhine-Meuse-Scheldt estuary (28). We generated a year-long time series of metatranscriptomic data which elucidate the unique functional properties of various ecosystem states and reveal typical metabolic traits of dominant plankton groups. Additionally, we investigate key features of the Southern Bight plankton assemblages such as the relationship between biodiversity and functional richness, the spatial variation resulting from the mixing of oceanic and estuarine waters, and seasonal shifts in feeding modes of certain microeukaryote species.

## RESULTS

### The Belgian North Sea

**Fig 1:**
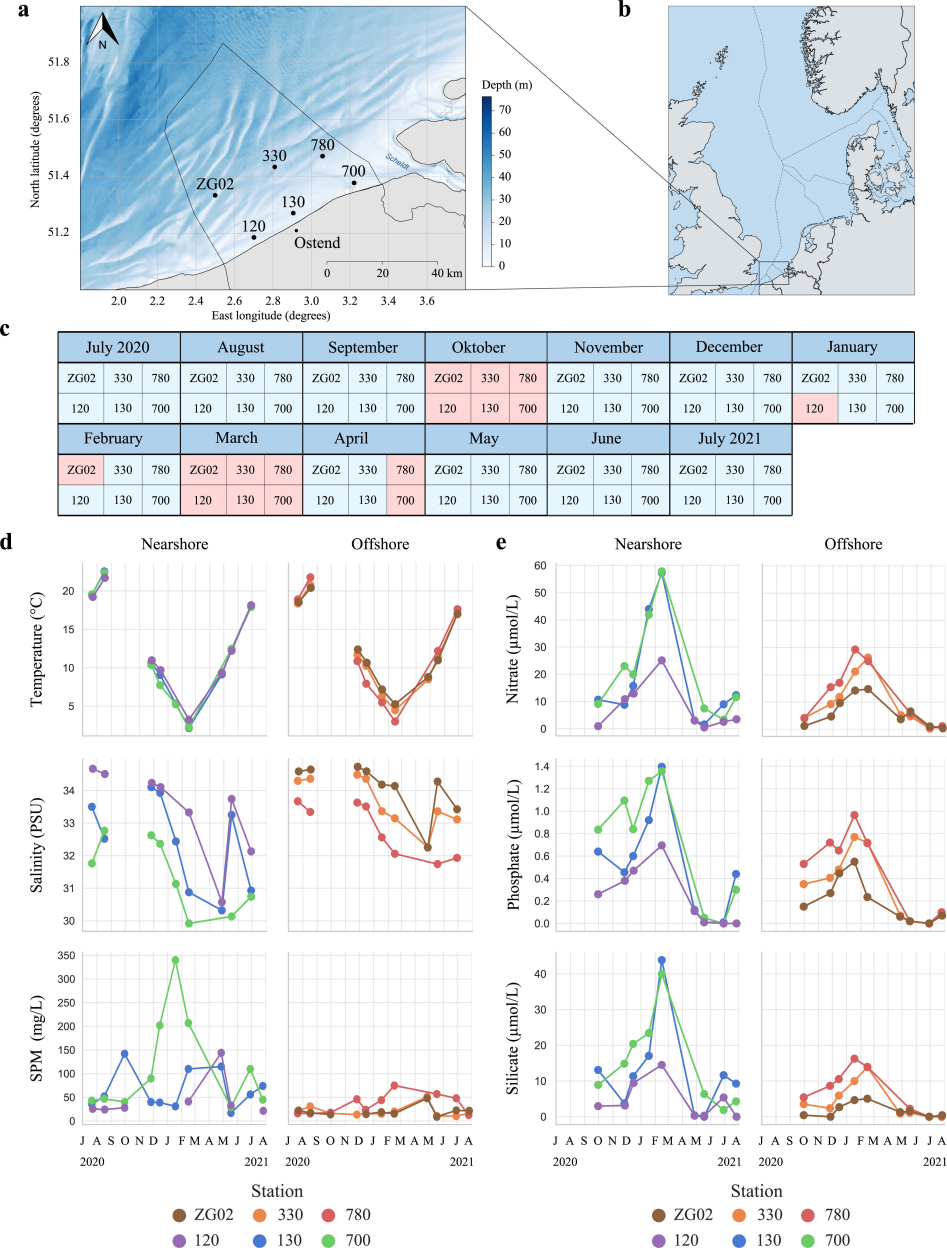
Sampling stations and their temporal and oceanographic context. (**a**) Geographical location of the six sampling locations in the Belgian North Sea (30). (**b**) Location of the sampling area in the wider North Sea region (map by NordNordWest/Wikipedia, distributed under a CC-BY-SA-3.0-DE license). (**c**) From July 2020 to July 2021, metatranscriptomic data were generated from these six stations. Gaps (in red) are due to canceled campaigns because of stormy weather conditions or coronavirus disease 2019 measures. For October 2020 and March 2021, weather conditions were too harsh to visit any station. **(d**) Seasonal changes in temperature, salinity, and suspended particulate matter (SPM) for near- and offshore stations. (**e**) Seasonal changes in nitrate, phosphate, and silicate nutrient levels for near- and offshore stations.

Six locations in the Belgian North Sea were sampled monthly to construct time series of oceanographic, meteorological, nutrient, pigment, biotic, and metatranscriptomic data (see Materials and Methods; Table S1; Fig. 1). Water temperature in the southern North Sea measured between 2.17°C and 22.54°C in winter and in summer, respectively. Nutrient concentrations rose over winter, reaching the highest values in February, and were depleted by April (Data Set S1). Suspended particulate matter (SPM) and salinity also varied, with fresher and more turbid water in winter, and more saline and clearer water in summer/fall. Spatially, levels of SPM, nitrate, phosphate, and silicate differed across locations, with stations 130 and 700 often showing the highest concentrations (Fig. 1; Data Set S1). Spatial differences in nutrient load and salinity in the study area are structured according to proximity to the Scheldt estuary in the east, with its freshwater discharge decreasing salinity and increasing nutrient levels in the Belgian coastal waters, while the inflowing North Atlantic water through the English channel from the west buffers temperature variations (29).

### Phenology of taxonomic groups

**Fig 2:**
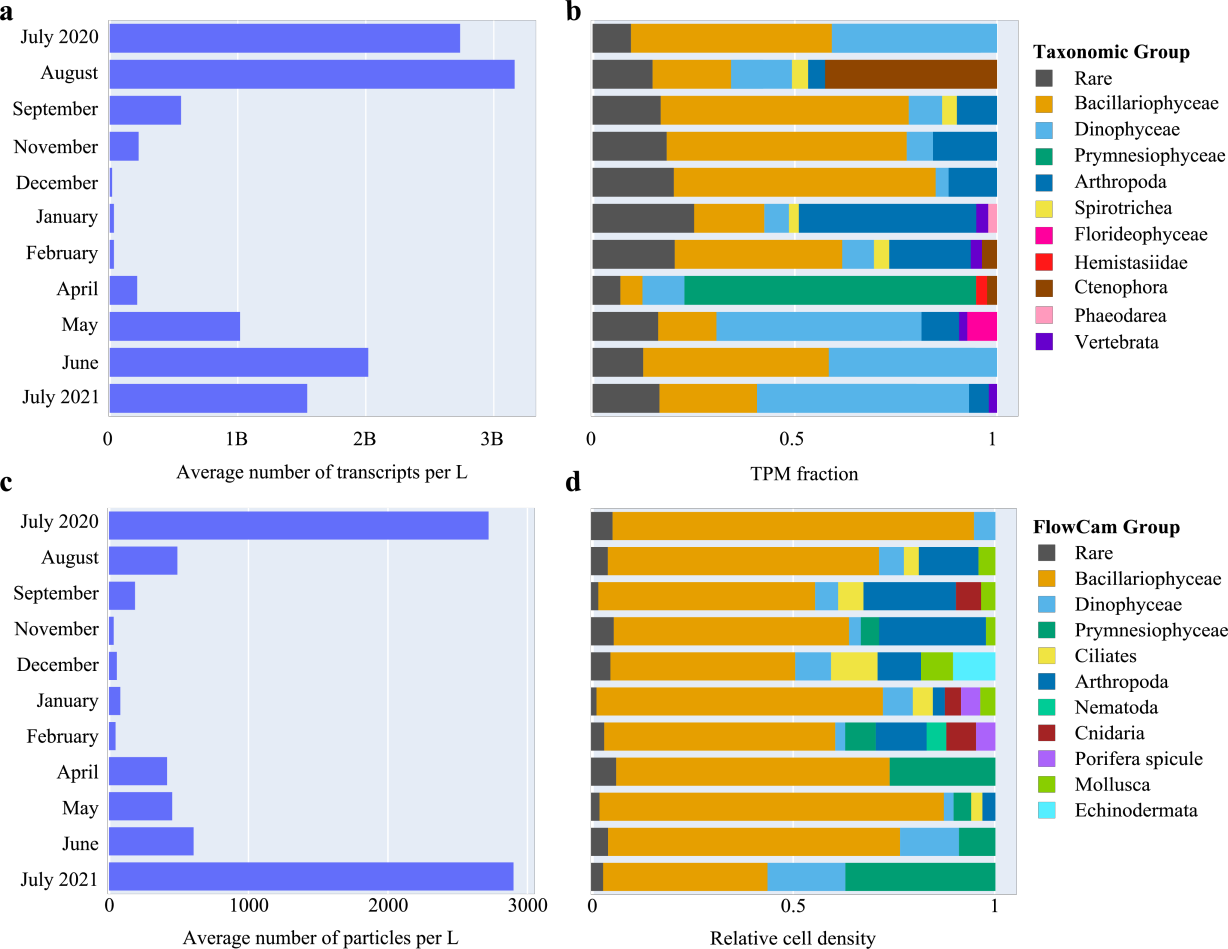
Monthly biomass fluctuations and turnover in taxonomic composition. (**a**) Monthly estimates for the number of extracted transcripts per liter of sea surface water, averaged across sampling stations. (**b**) Monthly relative transcript abundance fraction of high-level taxonomic groups annotated using EukProt (>60% sequence identity with reference), averaged across stations. The relative abundance fraction of a group in a given month was calculated as the sum of transcripts per million (TPM) annotated to that group divided by the TPM sum over all groups of that month (excluding unannotated transcripts). When the relative abundance fraction of a group was <2%, it was labeled as “rare.” (**c**) Monthly number of particles per liter of seawater, averaged across stations, calculated using FlowCam automated image analysis (excluding unannotated particles). (**d**) Relative cell densities of taxonomic groups per month, averaged across stations, as observed through FlowCam automated image classification. When the relative cell density of a group was <2%, it was labeled as rare.

**Fig 3:**
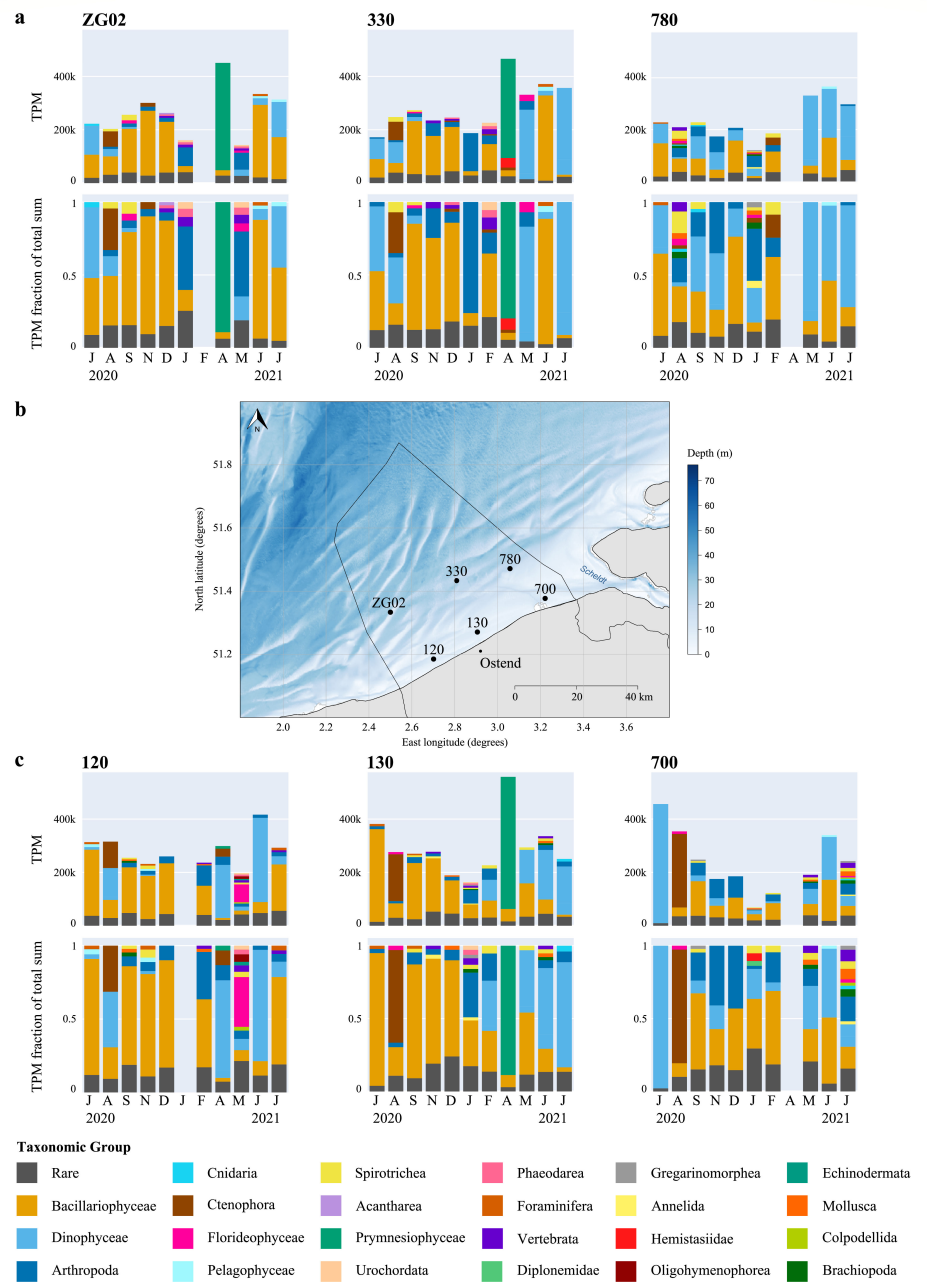
Monthly relative transcript abundance and relative taxonomic composition at each sampling station. (**a**) Monthly relative transcript abundance (TPM, top panels) and relative transcript abundance fraction (bottom panels) for taxonomic groups annotated using EukProt, per offshore sampling station. The relative transcript abundance represents the sum of TPM belonging to a taxonomic group. The relative abundance fraction of a group in a given month was calculated as the TPM sum of transcripts annotated to that group divided by the TPM sum over all groups for that month (excluding unannotated transcripts). When the relative abundance fraction of a group was <2%, it was labeled as “rare.” (**b**) Spatial location of the six sampling stations in the Belgian North Sea. (**c**) Monthly relative transcript abundance (top panels) and relative transcript abundance fraction (bottom panels) for taxonomic groups annotated using EukProt, per nearshore sampling station. Relative transcript abundances and fractions were calculated as for the offshore stations.

Our metatranscriptomic analysis yielded over 1.049 billion raw reads and 7 million unique transcripts (see Fig. S1 and details in the supplemental information). Diatoms were present year-round (Fig. 2) but different diatom assemblages succeeded each other (Fig. S2 and S3). The prymnesiophyte algae *Phaeocystis* bloomed in April and was detected in three of the four stations visited that month (Fig. 3). From May to July, the microeukaryote ecosystem was characterized by high relative abundances of dinoflagellate transcripts (Fig. 2 and 3), with *Noctiluca* accounting for the highest fraction of dinoflagellate transcripts (Fig. S4 and S5). In fall and winter, arthropods were relatively more abundant. Some taxonomic groups only occurred in certain months, e.g., high ctenophore abundances (*Mnemiopsis leidyi*), were found in August (Fig. S6). Microplanktonic biomass, determined as cell densities per liter of seawater using FlowCam automated microscopy, peaked in July (Fig. 2c). The estimated transcripts per liter (TPLs; see supplemental information) varied considerably between seasons and stations (Fig. S6). These “absolute” abundance estimates allow better assessment of differences in abundances and activity of specific taxa across samples than the relative abundance measure “transcripts per million” (TPM), but both measures are similarly biased by varying cell lysis and RNA extraction efficiencies across taxa and potential biases during other steps of sample processing such as filtering (see Materials and Methods) (31, 32). TPL is therefore not to be taken as a truly absolute expression measure and can only be used to compare expression levels across samples for taxa with similar biases in terms of lysis and RNA extraction efficiency (Fig. S6 through S8).

The spatial variability in taxonomic composition and relative transcript abundance across the six stations is substantial (Fig. 3). For example, we detected *Hemistasia*, a flagellate predator of *Phaeocystis* and other protists (33), in only one of the three stations that had a *Phaeocystis* bloom. Diatoms exhibited lower relative abundance in autumn at stations 780 and 700 (Fig. 3), and in absolute (TPL) terms, high diatom abundances were found in July and August 2020 at stations 120 and 130 (Fig. S6 and S7). Distinctive blooms were observed at stations ZG02 and 330 in June 2021 (Fig. S6), likely of the genus *Guinardia* (Fig. S3). Nearshore stations 120, 130, and 700 exhibited substantially higher TPL levels for ctenophore transcripts in August 2020 than the offshore stations (Fig. S6). The observed spatial differences likely reflect geographical and hydrodynamic differences between the sampled stations (such as depth, distance to shore, and the presence of a mixing zone where inflowing North Atlantic waters meet outflowing Rhine-Meuse-Scheldt waters) and associated differences in, e.g., water temperature, nutrient load, and salinity.

### Exploring functional diversity

Absolute transcript abundance was found to be highest when FlowCam-derived cell abundance levels were at their peak (Fig. 2, details in supplemental information).

Functional richness, however, quantified as the number of unique enzymatic functions [Kyoto Encyclopedia of Genes and Genomes Orthology (KEGG KO) identifiers] present in a sample, did not differ between months [Fig. S9; analysis of variance (ANOVA), *F*(10,51) = 1.062, with degrees of freedom 10 and 51 representing #groups −1 and #samples − #groups, *P* = 0.40]. Similarly, no significant differences were observed between stations [ANOVA, *F*(5,56) = 0.594, *P* = 0.704] or when considering the number of unique protein families (PFAM) instead [Fig. S10; ANOVA_months_, *F*(10,51) = 0.896, *P* = 0.544; ANOVA_stations_, *F*(5,56) = 0.731, *P* = 0.604]. Principal component analysis of KEGG KO relative expression profiles (TPM), however, revealed temporal and spatial variations in the relative expression of metabolic functions in the microeukaryotic ecosystem across sampling months and stations (Fig. S9a). The functional dissimilarity between seasons coincides with distinct nutrient and temperature regimes, while the differences within summer months correlate with differences in suspended particulate matter load (Fig. S9; Fig. 1a).

Active species richness averaged across stations was higher from September to February and lower from May to July [Fig. S11; ANOVA, *F*(10,51) = 9.872, *P* = 6.185 × 10^−9^]. No strong correlation was found between functional richness and the number of active species [Pearson’s correlation (PC), *r* = 0.18, df = 60, *P* = 0.151]. A negative correlation was found between log-transformed FlowCam cell abundances and the number of active species (*r* = −0.43, df = 60, *P* = 0.0008). Likewise, there is a weak negative correlation between functional richness and log-transformed FlowCam cell abundances (*r* = −0.28, df = 55, *P* = 0.036). These negative correlations likely arise from non-homogeneous distribution of species in high biomass situations, which impacts the detection of rare species with the given sequencing depth (34) (Fig. S11).

In conclusion, from September to February, there is an increase in the number of metabolically active species. This increase, however, does not result in increased functional richness.

### Functional seasonal dynamics of microeukaryotic plankton

**Fig 4:**
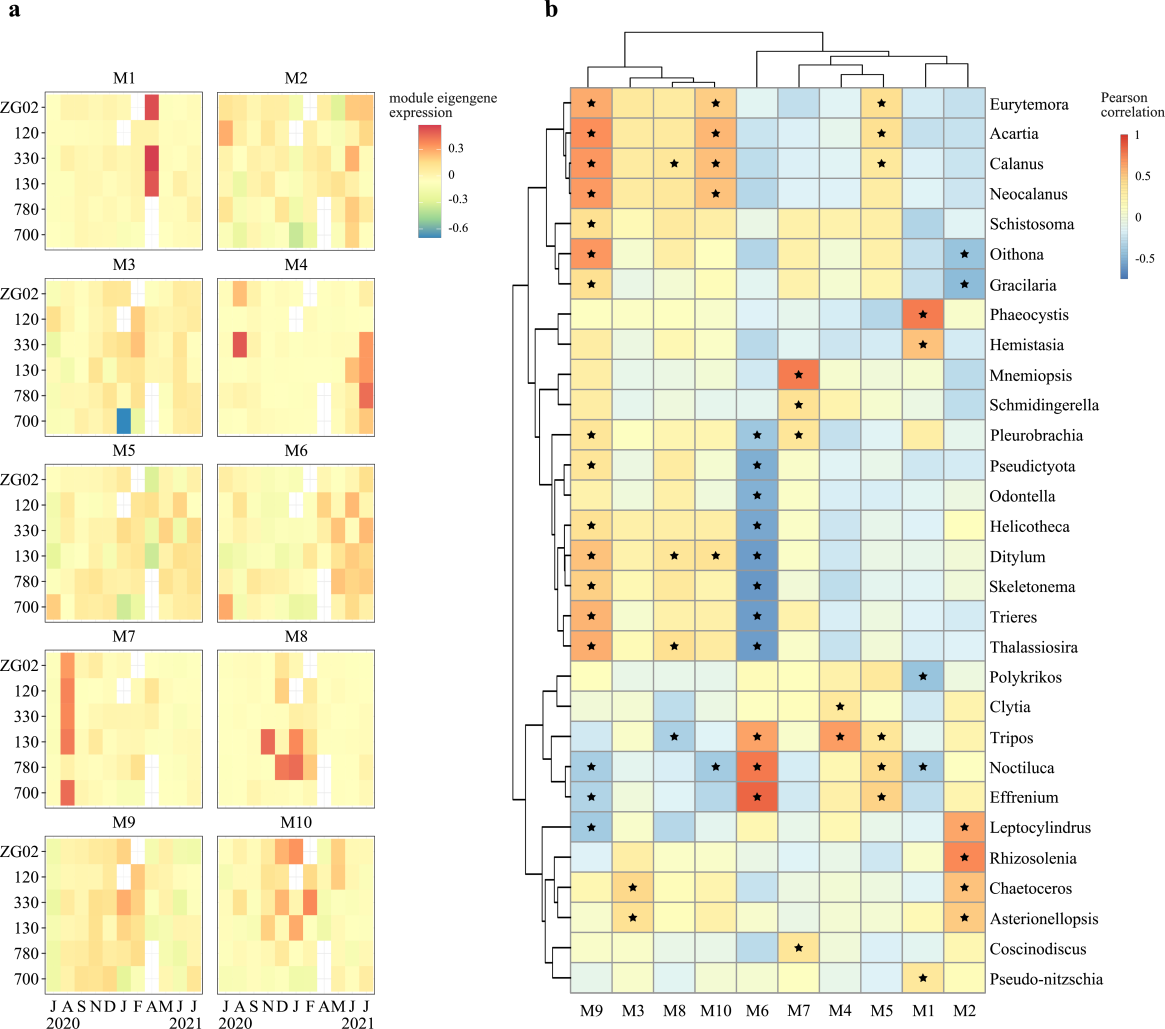
Module eigengene expression and correlation with taxonomic groups. (**a**) Heatmap of module eigengene expression levels across samples, ordered by station and month of sampling. Eigengenes represent the first principal component of a module. Modules were calculated using weighted gene correlation network analysis on TPM expression values of KEGG KO identifiers. (**b**) Pearson correlation of module eigengene expression per sample with log-transformed relative genus abundances of EukProt-annotated transcripts (TPM sums per sample). Black stars indicate correlations with *q* values (Benjamini and Hochberg adjusted *P* values) of <0.05.

To get a better understanding of how metabolic functions vary and co-vary in the Southern North Sea microeukaryotic plankton assemblages and how this is related to the presence of particular taxa, we applied weighted gene co-expression network analysis (35) to the KEGG KO relative expression profiles across samples. Ten distinct modules of co-expressed functional identifiers were identified (Fig. 4). Analysis of module eigengene expression revealed fall and winter clusters (M3, M8, M9, and M10) that are correlated with the relative transcript abundance of copepods and/or certain diatom genera, and spring/summer clusters that represent different ecosystem states: a *Phaeocystis* bloom in April (M1), a dinoflagellate dominance from May to August (M4–M6), diatom assemblages in June and July (M2), and the occurrence of *Mnemiopsis* in August (M7) (Fig. 4). For each module, we then determined characteristic KEGG pathways that were strongly represented in the module (see Materials and Methods) and quantified the contribution of the genus whose absolute abundance was most strongly correlated to the module’s eigengene expression to the overall expression of these pathways.

Module M1 eigengene expression predominantly correlates with increased relative transcript abundance of the prymnesiophyte genus *Phaeocystis* (Fig. 4b). KOs involved in cell cycle control and lysine and brassinosteroids biosynthesis displayed significantly higher ranked correlations with M1 eigengene expression than KOs associated with other pathways (Data Set S2). For lysine biosynthesis and cell cycle control, most taxonomically annotated transcripts that were expressed during the April *Phaeocystis* bloom were indeed annotated to *Phaeocystis* (Fig. S12; Data Set S2 and S3). None of the transcripts associated with brassinosteroids biosynthesis were annotated as *Phaeocystis*. This indicates that either the *Phaeocystis* reference transcriptome did not include genuine *Phaeocystis* genes involved in the biosynthesis of brassinosteroids, or that another unidentified organism produces brassinosteroids during a *Phaeocystis* bloom. Brassinosteroids have been associated with growth, development, and protection against abiotic stress in plants and could have similar functions in *Phaeocystis* (36).

The module M2 eigengene profile exhibits a correlation with a spring/summer diatom assemblage, mainly with the genera *Rhizosolenia*, *Leptocylindrus*, *Chaetoceros*, and *Asterionellopsis* (Fig. 4). Functionally, this module contains KOs involved in the biosynthesis of amino acids and co-factors, biotin, and vitamin B6 metabolism (Fig. S12; Data Set S2). Additionally, the identification of pathways related to porphyrin metabolism may imply regulation of pigments in response to light and nutrient availability. Taxonomic attribution of these pathways’ expression indicates a substantial relative diatom contribution in fall, but not in spring, likely because *Guinardia* was not included in the EUKprot reference database (Fig. S12). Furthermore, the module predominantly encompasses pathways that are broadly expressed over time, indicating that their presence is not exclusive to this module or its associated diatom assemblage.

Eigengene expression of modules M3 and M8–M10 is correlated with the absolute abundance of a diverse range of genera, from copepods to diatoms. These modules encompass KOs from a diverse suite of pathways linked to fundamental aspects of (multicellular) cell growth, regulation, and maintenance such as ribosome synthesis and mRNA surveillance, signaling (based on transcripts homologous to genes involved in, e.g., the ErbB and Hippo signaling cascades in mammals) and synthesis of glycolipids and glycosaminoglycans, vital for cell adhesion and communication and as structural components. Moreover, the presence of pathways associated with responses to bacterial and viral infections suggests an important role of host defense (Fig. S13 through S16; Data Set S2 and S3).

Module M6 eigengene expression, peaking in late spring and summer, correlates with the relative (and absolute) transcript abundance of dinoflagellate genera such as *Noctiluca*, *Tripos*, and *Effrenium*. Module M6 is linked to the synthesis of acarbose and validamycin, suggesting a capacity for antimicrobial compound production. Furthermore, module M6 is associated with the degradation of various aromatic compounds, emphasizing the dinoflagellates’ role in organic matter breakdown and heterotrophic activities. Particularly noteworthy is the involvement of pathways related to the degradation of benzoate, flavonoids, and limonene, indicating the potential recycling of xenobiotic metabolites in sea surface waters by these assemblages. Additionally, module M6 is associated with metabolic functions encompassing vitamin B6 synthesis, polyketide sugar unit biosynthesis, biosynthesis of various plant secondary metabolites, and teichoic acid biosynthesis. Transcripts involved in these pathways were confirmed to be expressed by *Noctiluca* (Fig. S14).

Module M7 eigengene expression was strongly correlated with increased relative (and absolute) transcript abundances of the comb jelly genus *Mnemiopsis* and peaked in all stations (except 780) in August. This module is associated with homologs of mammalian genes involved in the ErbB signaling pathway, natural killer cell-mediated cytotoxicity, and bacterial invasion of epithelial cells (Data Set S2). While these pathways are detected across all months, a large part of the expression of these pathways in August could be attributed to *Mnemiopsis* (Fig. S13). Enzymes within these pathways are likely involved in either defense against microorganisms or the mechanisms of predation deployed by *Mnemiopsis*.

Lastly, module M4, with peak eigengene expression in summer, was associated with the degradation of limonene and flavonoids. Module M4’s eigengene expression showed a correlation with the relative transcript abundance of the dinoflagellate genus *Tripos* and the cnidarian genus *Clytia*. Transcripts involved in these pathways were confirmed to be expressed by *Tripos* (Fig. S13) and *Noctiluca* (Fig. S14) with expression profiles peaking in summer, even though relative transcript abundances of *Noctiluca* did not significantly correlate with M4 eigengene expression (Fig. 4; Data Set S3). Both genera are thus likely capable of catabolizing biotic carbon sources. Modules and their contents can be further explored in the supplemental data (Data Set S2).

### The case of dinoflagellates, diatoms, and *Phaeocystis*

We focused on the most abundant taxonomic groups to gain a better insight into how functional richness links to the number of active species and how this relates to their ecology. The overall relatively most abundant microeukaryote groups detected in the metatranscriptome were diatoms, dinoflagellates, and the prymnesiophyte *Phaeocystis globosa* (Fig. 2).

From September to December, we observed higher relative abundances of diatom transcripts (Fig. S2 and S3) accompanied by a significant increase in the number of active diatom species [Fig. S17; Kruskal-Wallis, *H*(10,51) = 38.003, *P* = 3.791 × 10^−5^]. The fall assemblage was dominated by the genera *Trieres*, *Ditylum*, *Thalassiosira*, *Odontella*, and *Helicotheca*. A smaller peak in relative diatom abundance [but a large peak in estimated absolute abundance (TPL), Fig. S6] was observed at stations ZG02 and 330 during June and July 2021, where the community was mainly composed of *Guinardia* (based on PhyloDB, Fig. S3). Stations closest to the Scheldt estuary, i.e., 700 and 780, exhibited the lowest relative diatom abundances. Diatom communities showed clear temporal structuring, aligning with temperature, and spatial gradients, including variations in nutrients, salinity, and SPM levels (Fig. S17d). For example, the eigengene expression of module M9, a set of KOs that correlates with the abundance of a *Thalassiosira* dominated diatom community, correlates significantly with elevated nutrient levels [Fig. 4; Fig. S18; NO_3_^−^ *r*(10) = 0.32, *P* = 0.01; PO_4_^3−^ *r*(10) = 0.36, *P* = 0.004; Si *r*(10) = 0.27, *P* = 0.04]. The diatom genera that correlate most with module M9 eigengene expression are most abundant at stations 130, 120, 330, and ZG02 (Fig. S2).

**Fig 5:**
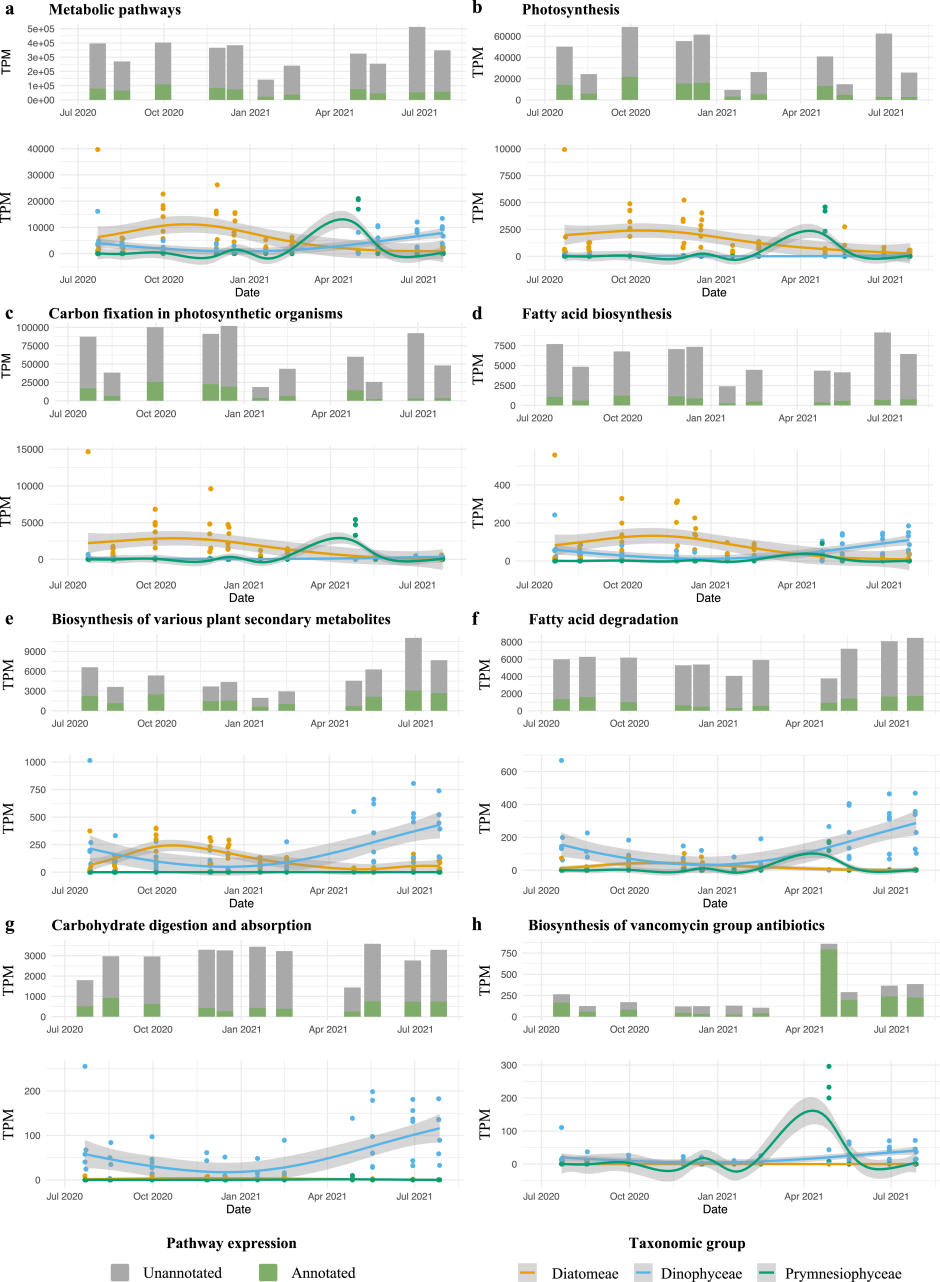
Expression of selected pathways and generalized additive model (GAM) regression fits for the expression of diatom, dinoflagellate, or *Phaeocystis* transcripts. Each panel shows the TPM expression profiles of transcripts associated with a given KEGG pathway of interest (top figures), alongside GAM regression fits to the summed TPM expression profiles of pathway transcripts that were annotated to diatoms, dinoflagellates, or *Phaeocystis* (>90% sequence identity) (bottom figures). The top figure in each panel contrasts the summed TPM expression levels of taxonomically annotated versus unannotated transcripts associated with the KEGG pathway concerned. The lines are GAM fits, with the shaded areas representing the 95% confidence intervals for the smoothed mean estimates. Eight pathways are displayed: (**a**) “metabolic pathways,” an umbrella term containing all metabolic activities; (**b**) photosynthesis; (**c**) carbon fixation in photosynthetic organisms; (**d**) fatty acid biosynthesis; (**e**) biosynthesis of various plant secondary metabolites; (**f**) fatty acid degradation; (**g**) carbohydrate digestion and absorption; and (**h**) biosynthesis of vancomycin antibiotics.

The diatom community’s functional richness appeared relatively consistent throughout the year and scales with the number of species present [Fig. S17c; linear model: MSE = 236,859.62 (SD = 103,071.89), *R*^2^ = 0.3044 (SD = 0.1924); second-degree polynomial model: MSE = 279,665.89 (SD = 130,398.46), *R*^2^ = 0.1720 (SD = 0.3299); a paired *t*-test revealed no statistically significant difference between the performance of the two models, *P* = 0.466]. In the fall, diatoms contributed most to the metabolic activity of the microeukaryotic plankton, exhibiting the highest relative expression of transcripts involved in primary production processes such as photosynthesis, carbon fixation, and fatty acid biosynthesis (Fig. 5; Fig. S19). Predictions from the trophic mode classifier model by Lambert et al. (37) consistently identified diatom species bins as autotrophic (Fig. S20 and S21; see Materials and Methods), although predictions were not feasible for smaller transcriptome bins.

Dinoflagellates showed peak relative and absolute abundance from May to July (Fig. 3; Fig. S8), with *Noctiluca* being the most abundant genus (Fig. S4 and S5). The functional profiles of dinoflagellates varied predominantly along a temperature gradient (Fig. S17h). Module 6, associated with *Noctiluca*, also positively correlated with temperature (Fig. 4; Fig. S18; *r* = 0.27, df = 10, *P* = 0.03), underscoring a seasonal trend in dinoflagellate abundance and activity. Notably, *Noctiluca* persisted in the colder, nutrient-rich, and less saline waters of stations 780 and 700 during fall and winter months (Fig. S4 and S5). Few active dinoflagellate species were detected across samples (Fig. S17). However, one to three active species of dinoflagellates can display a wide variety of functions, with maximum values that exceed double the number of diatom functional richness. Intriguingly, the Lambert et al. model (37) predicted the EukProt-annotated *Noctiluca scintillans* taxonomic bin as consistently phototrophic (Fig. S20), while the PhyloDB annotated *Noctiluca scintillans* bin was predicted to be heterotrophic during the summer months, from May to September, and phototrophic in other months (Fig. S21). Similarly, based on PhyloDB annotations, *Tripos fusus* was predicted as heterotrophic in July 2021 and phototrophic during other months (Fig. S21), whereas the EukProt annotated *Tripos* bin was consistently predicted as phototrophic (Fig. S20). Very few transcripts involved in photosynthesis were linked to dinoflagellates, while they do express transcripts involved in autophagy, fatty acid degradation, and carbohydrate digestion and absorption, among others (Fig. 5; Fig. S19; Data Set S3). The observed dinoflagellates’ ability to rely on both photo- and heterotrophy may contribute to the higher functional richness observed in dinoflagellate transcriptomes.

The prymnesiophyte *Phaeocystis globosa* bloomed in April, dominating the microplankton community in the Southern Bight in both relative and absolute abundance and metabolic activity (Fig. 2 and 3; Fig. S6). This bloom did not show high TPL values, potentially due to the lower amount of seawater filtered due to clogging filters (Data Set S1). The spring *Phaeocystis* bloom did not significantly correlate with distinct temperature, salinity, or nutrient values (module M1 in Fig. S18). The Lambert et al. (37) trophic model predictions based on both PhyloDB and EukProt annotation sources indicated that these *Phaeocystis* blooms were heterotrophic (Fig. S20 and S21). However, they also expressed transcripts involved in photosynthesis and carbon fixation (Fig. 5). Furthermore, we found that *Phaeocystis* was a major contributor to the expression of genes involved in specific pathways, such as the α-lipoic acid metabolism, the biosynthesis of polyketide sugar units, and the biosynthesis of antibiotics (Fig. 5; Fig. S19). Surprisingly, for samples that contained *Phaeocystis* blooms, we found very high expression of genes involved in certain pathways that were not annotated as *Phaeocystis*, e.g., zeatin biosynthesis or flagellar assembly. These might constitute vital processes in the *Phaeocystis* life cycle that are currently not represented in the reference transcriptome, e.g., an investment in motile microflagellate cells after the blooming phase (38) (Fig. S19).

High relative abundance of diatoms, dinoflagellates, or *Phaeocystis* is often accompanied by a high relative expression of genes related to viral infection, e.g., homologs of genes involved in pathways related to coronavirus and papillomavirus infections in humans (Fig. S19). This indicates that higher abundances of these taxa are associated with a heightened viral activity or prevalence, which suggests that viral infections may be a significant factor in microeukaryotic population dynamics (39).

## DISCUSSION

The observed seasonal shifts in taxonomic assignments of gene transcripts echo well-documented phenological patterns in the Southern Bight. These include the early spring bloom of *Phaeocystis globosa* (40), the prevalence of *Noctiluca scintillans* between May and July (41), and the late summer bloom of the invasive ctenophore *Mnemiopsis leidyi* (42). These phenological patterns are intricately linked to the seasonal oscillations of abiotic factors such as temperature, nutrient availability, or solar irradiance. Our study further reveals seasonal partitioning of key metabolic pathways among the predominant groups. For example, we observed a shift between diatoms and dinoflagellates alternating in their contribution to the production of fatty acids and other secondary metabolites.

The first plankton bloom of the year in the Southern Bight occurs in diatoms in spring, leveraging the elevated temperatures and sunlight to utilize the accumulated winter nutrients derived from river discharge, atmospheric deposition, and sediment resuspension (29, 43). We observed a rise in metabolic activity of diatoms already in February, preceding the actual increase in cell densities detected by imaging in spring. After the first diatom bloom, *Phaeocystis globosa* capitalizes on favorable light and nutrient concentrations and forms colonies that heavily invest in primary production (44, 45). Recent laboratory studies have shown that these *Phaeocystis* blooms attract a specialized bacterial community (46). Our *in situ* findings suggest that *Phaeocystis* implements a heterotrophic strategy at later stages of the bloom. This might represent an adaptive strategy where *Phaeocystis* cultivates and attracts bacteria during favorable periods and subsequently feeds on them following bloom senescence and nutrient depletion. While we did not find clear evidence of mixotrophy for *Phaeocystis* in trophic mode predictions, we observed the expression of transcripts involved in both photosynthesis and heterotrophy (45).

*Noctiluca scintillans* likely deploys different feeding strategies across seasons, potentially underpinning their ecological success. The pronounced functional richness of dinoflagellates confirms an elevated level of metabolic flexibility, a hypothesis supported by the ambiguous trophic mode predictions and the presence of degradation, digestion, and absorption pathways. This versatility potentially confers a competitive edge in fluctuating environments (47).

From summer to fall, diatoms exhibited higher abundances and increased expression of transcripts involved in primary production processes such as photosynthesis, carbon fixation, and fatty acid biosynthesis.

The increased expression of antimicrobial or immunity-related pathways during blooms of *Phaeocystis globosa* and *Mnemiopsis leidyi* points toward the critical role of biotic interactions in shaping community dynamics (48). The occurrence of transcripts associated with the production of antibiotics in *Phaeocystis* suggests a mechanism by which the *Phaeocystis* colonies could defend against a *Hemistasia* infection (33). These observations align with the growing consensus on the importance of parasites and pathogens in regulating bloom events (49).

Our study demonstrates the value of metatranscriptomics data to characterize the dynamics of taxonomically resolved metabolic functions during the seasonal succession of planktonic communities. In microbial communities, such metabolic functions are important predictors of the food web position and ecological role of the individual species (50). Facing unprecedented environmental perturbations due to anthropogenic change, knowledge of the natural dynamics of metabolic functions of individual taxa will be critical to understanding and ultimately predicting the stability and resilience of planktonic communities.

## MATERIALS AND METHODS

### Sampling

The data presented in this study were obtained from seagoing campaigns from July 2020 to July 2021. Several stations in the Belgian North Sea were sampled every month with the R/V Simon Stevin (51, 52) (offshore stations: ZG02, 330, and 780; nearshore stations: 120, 130, and 700; see Fig. 1; Data Set S1). When at sea, navigation, meteorological, and oceanographic data were measured using the ship’s underway system. At each sampling station, temperature (Celsius) and depth (meter) profile (CTD) data were collected with a Seabird SBE25plus CTD (Sea-Bird Scientific, Bellevue, WA USA). Additionally, the following parameters were quantified: salinity of the water body, expressed in practical salinity units, dissolved oxygen, pH, Secchi depth, electrical conductivity of the water, density of the water body, fluorescence of the water body, optical backscatter as turbidity of the water body, photosynthetic active radiation, pressure, and the concentration of SPM in the water body (expressed in milligram per liter) (Data Set S1). Water samples were taken with Niskin bottles at a depth of 3 m and were analyzed for nutrient and pigment concentrations. For more detail on sampling procedures, see Mortelmans et al. (51).

Microphytoplankton biomass was estimated using FlowCam analysis, which involved collecting 50 L of sea surface water, filtering, and processing with a FlowCam VS-4 benchtop model (Fluid Imaging Technologies, Yarmouth, Maine, USA) to classify particles into distinct taxonomic groups; further details are provided in the Supplemental Methods and Martínez et al. (11).

Samples for metatranscriptomic analysis were collected as follows: at each sampling location, 50 L of sea surface water was manually collected with stainless-steel buckets and filtered through two stacked stainless-steel sieves with mesh sizes of 250 and 50 µm. In case of extreme bloom events, smaller volumes were filtered due to the high biomass clogging the filters. From the 50-µm sieve, the collected residue was resuspended in 9–45 mL of seawater, with the eluate volume depending on the residue biomass (Data Set S1). The samples were then stored in cryovials and flash-frozen in liquid nitrogen within 5 minutes of collection. Back in port, samples were transferred to a −80°C ultra-freezer until RNA extraction.

### Extraction and quality control of RNA, library preparation, and sequencing of cDNA

RNA was extracted with the RNeasy Mini Kit (Qiagen), according to a modified version of the manufacturer’s protocol. Briefly, samples were thawed, centrifuged, and the supernatant seawater was removed. Lysis buffer with 700 mg of RNA-free silica beads (350 mg each of 0.1 and 0.5 mm) was added to the sample, after which samples underwent two cycles of homogenization and cooling on a metal cooling block (−20°C) for 1 minute each. The RNA was then extracted using the kit’s spin columns. Depending on the season and biomass of microplankton, different starting volumes were used for RNA extraction (Data Set S1). The extracted RNA was eluted in 52 µL of RNAse-free water and stored in the −80°C freezer. Two microliters of the total RNA eluate was used for quality and yield analysis. Total RNA yield was measured using a Qubit fluorometer (Invitrogen), RNA quality was assessed using a Bioanalyzer (Agilent). After extraction, samples were shipped to the VIB Nucleomics core facility (https://nucleomicscore.sites.vib.be/) on dry ice. There, RNA concentration and purity were determined spectrophotometrically using the Nanodrop ND-8000 (Nanodrop Technologies), and RNA integrity was assessed using a Bioanalyzer 2100 (Agilent). A standard volume of external RNA controls consortium (ERCC) spike-in mix was added before starting the library preparation (but after lysis, extraction, and RNA yield measurement, allowing us to maintain an ERCC spike-in/total RNA proportion around 1%, thereby avoiding overwhelming the sequencing libraries with spike-in sequences). Per sample, an amount of 200 ng of total RNA was used as input. Using the Illumina TruSeq Stranded mRNA Sample Prep Kit (protocol version: # 1000000040498 v00 October 2017), poly-A containing mRNA molecules were purified from the total RNA input using poly-T oligo-attached magnetic beads. In a reverse transcription reaction using random primers, RNA was converted into first-strand cDNA and subsequently converted into double-stranded cDNA in a second-strand cDNA synthesis reaction using DNA polymerase I and RNAse H. The cDNA fragments were extended with a single “A” base to the 3′ ends of the blunt-ended cDNA fragments, after which multiple indexing adapters were ligated, introducing different barcodes for each sample. Finally, enrichment PCR was carried out to enrich those DNA fragments that have adapter molecules on both ends and to amplify the amount of DNA in the library. Sequence libraries of each sample were equimolarly pooled and sequenced on Illumina NovaSeq 6000 [SP300 flow cell, PE150 (151-8-8-151)] at the VIB Nucleomics Core.

### Preprocessing, assembly, and translation

Raw sequences were inspected using FastQC and MultiQC (53). Sequencing adapters were trimmed from the reads using Trimmomatic (54) (version 0.39, parameters: ILLUMINACLIP:ADAPTERS:2:30:7 LEADING:2 TRAILING:2 SLIDINGWINDOW:4:2 MINLEN:50). Ribosomal RNA sequences were removed using RiboDetector (55). Quality-controlled, trimmed non-rRNA PE reads were assembled in nucleotide space using rnaSPAdes (version 3.15.3, parameters: --rna -k 75,99,127) (56). All assemblies were then combined and clustered at 95% identity using MMseqs2 easy-linclust algorithm (version 13–45111) (46, 57). The contig names of the resulting metatranscriptome were standardized with anvi-script-reformat-fasta from Anvio (version 7.1) to reduce their size and facilitate subsequent analysis (58). Assembled transcripts that matched the ERCC92 spike-in RNA sequences were identified using MMseqs2 easy-search and removed, resulting in the final metatranscriptome (57). Protein coding regions in the *de novo* assembled transcripts were identified by Transdecoder (version 5.5.0, parameters: --single-best-only) (59).

### Transcript annotation and quantification

To assign taxonomic information to the assembled and translated sequences, they were searched against EukProt (20) and an extended version of PhyloDB (version 1.075; https://allenlab.ucsd.edu/data/), using MMseqs2 (57). The PhyloDB database was extended with sequence data from ENA for species that were identified in FlowCam data but were not included in the database (60). If a published shotgun assembly transcriptome existed for that species, it was added to the reference database. For both databases, best alignment hits with a sequence identity of >90% or >60% (>60% sequence identity when considering taxonomic classes) were retained for subsequent analysis (27, 61). Peptide translations of the *de novo* assembly were functionally annotated using the eggNOG reference database (62) and the eggNOG-mapper tool (version 2.1.7) (63). Transcript and ERCC spike-in standards were quantified using Kallisto, yielding TPM counts for every transcript (64). The relative abundance fraction of a given taxonomic group was calculated as the sum of all TPM values belonging to that level divided by the total TPM sum of all taxonomically annotated transcripts for that sample or month, ignoring unannotated transcripts. Transcripts per liter were estimated by relating the TPM counts to the spike-in recovery values while accounting for sample processing volumes and RNA yield (27) (see Supplemental Methods).

### Statistical analysis

Principal component analyses were performed on pre-processed, log-transformed TPM values using sci-kit learn in python (version 1.1.3) (65, 66). Arrows representing the correlation between the first two principal components and environmental parameters were added to the plots.

A weighted gene co-expression network analysis was performed using the weighted gene correlation network analysis (WGCNA) package (35) (version 1.72–1) in R (65). The approach was modified from the analysis pipeline described in Cohen et al. (47). Briefly, we aimed to identify co-expression modules of KEGG KO identifiers based on log-normalized count sums across annotated transcripts. The expression matrix was pre-processed by setting TPM values below 1 to 0 and removing KOs with aggregate counts less than 10 across all samples. Outlier samples were detected using the WGCNA scaled connectivity measure, and sample dendrograms were constructed using average linkage hierarchical clustering on the PC network of KEGG KOs. After pre-processing, a correlation network was constructed from the expression matrix. The optimum number of modules was determined by exploring a range of soft-thresholding powers to ascertain the minimum value for which a scale-free topology was achieved, using the pickSoftThreshold function from the WGCNA package (Fig. S22). Module detection was performed using the WGCNA dynamic tree cut algorithm (minimum module size: 70, deepSplit = 4), and modules with highly similar expression profiles (PC >0.6) were merged. Module eigengenes, representing the first principal component of each module, were calculated using the WGCNA package moduleEigengene function, and their expression across samples and correlations with environmental parameters and the TPM abundances of taxonomic groups was assessed. Connectivity within modules was quantified using module membership, allowing for the pinpointing of functions that closely follow the module’s overall expression pattern. Module membership was defined as the Pearson correlation between module eigengene expression and KEGG KO identifier. To determine whether certain metabolic pathways were more associated with a highly connected gene function, we performed Mann-Whitney *U* tests to assess which pathways were more represented at the top of a ranked list of KEGG KO identifiers, ordered according to decreasing module membership, than expected by chance. Resulting *P* values were adjusted for multiple testing with the Benjamini-Hochberg procedure, which controls the false discovery rate (67, 68).

KEGG pathway expression was analyzed by collecting the total TPM expression of assembled transcripts that were annotated with a KO involved in that pathway. For these, we retrieved EukProt annotations with >90% sequence identity to be able to calculate the annotated and unannotated fraction or to assess which taxonomic group contributed most to the given pathway. For pathways identified as characteristic of modules identified through WGCNA, we visualized the total TPM expression of transcripts involved in each pathway and the fraction that could be assigned to the genus whose relative abundances correlated most with expression of the module eigengene. Generalized additive models (GAMs) were constructed with the mgcv package in R (version 1.8–40) to assess the temporal dynamics of gene expression for KEGG pathways (69, 70). To complement the GAM analysis, we aggregated the data by month and calculated the total TPM for each pathway, both for all transcripts and for transcripts that had a EukProt taxonomic annotation (>90% sequence identity). This was visualized alongside the GAM fits to provide a more comprehensive view of the pathway activity over time (71).

To obtain active species richness estimates of samples, taxonomic bins of transcripts with >90% sequence identity with sequences in reference databases were considered. Only taxonomic bins that had more than 100 non-zero expressed transcripts in at least one sample were retained, and their occurrence per sample was quantified. Functional richness was defined as the amount of unique KEGG KO identifiers found in a sample.

We used the classification machine learning model by Lambert et al. to predict the trophic mode of protist taxonomic bins (37). The training data, parameter settings, and selected features were used as in the original study (we implemented the XGBoost classifier with reported precision of 88% ± 10% in the original publication). Taxonomically annotated transcripts were binned to species level (>90% seq. id), and the model was trained with the MinMax-scaled MMETSP training data set from the original publication containing the union of selected features (37). Predictions were then generated for each sample by subsetting each profile to contain the union of selected features, imputing missing values with zeros, and scaling profiles with the pre-fitted MinMax scaler. Predictions could only be made for subsetted transcriptional profiles that contained >800 non-zero expressed PFAMs, as required by the Lambert et al. model (37). Monthly consensus predictions were determined by the majority vote of the individual samples’ predictions for the month.

## Data Availability

The metatranscriptomic data generated in this study are available on SRA under the BioProject PRJNA1021244. Analysis scripts are available on GitHub (https://github.com/MichielPerneel/BPNS_seasonal_MTX). All other data supporting the findings of this study are provided in the supplemental material.

## SUPPLEMENTAL MATERIAL

Data Set S1 mbio.00383-24-s0001.xlsx Environmental data, flowcam data, and sampling and lab metadata.

Data Set S2 mbio.00383-24-s0002.xlsx Pathways associated with modules of co-occurring KEGG KO identifiers.

Data Set S3 mbio.00383-24-s0003.xlsx Taxonomic associations with KEGG pathways.

Supplemental material mbio.00383-24-s0004.pdf Figures S1 to S23, Table S1, additional bioinformatic details, and additional methods.

ASM does not own the copyrights to Supplemental Material that may be linked to, or accessed through, an article. The authors have granted ASM a non-exclusive, world-wide license to publish the Supplemental Material files. Please contact the corresponding author directly for reuse.
