# Supplementary material for: Seasonal metabolic dynamics of microeukaryotic plankton: a year-long metatranscriptomic study in a temperate sea
Source: mBio. 2024 Jul 9;15(8):e00383-24. doi: 10.1128/mbio.00383-24 (PMC11323732; doi:10.1128/mbio.00383-24)
Supplement: Supplemental material — Figures S1 to S23, Table S1, additional bioinformatic details, and additional methods. [file mbio.00383-24-s0004.pdf]

Supplementary Information
Supplementary Figures

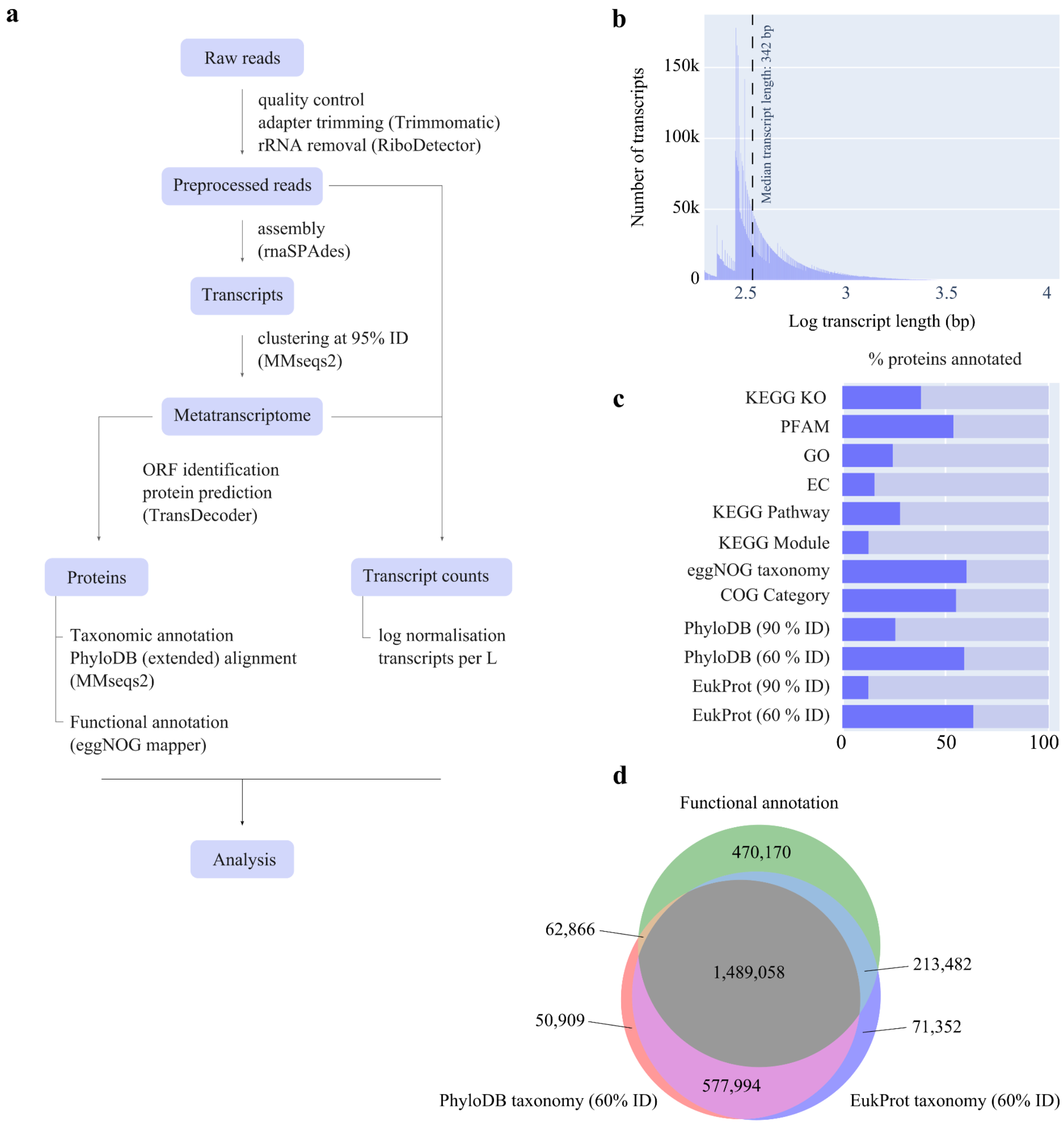

Supplementary Figure 1. Bioinformatics workflow and metatranscriptome metrics. a) Bioinformatics workflow. **b)** Log transcript length distribution in the de novo assembled metatranscriptome. The dotted line indicates the median transcript length (342 bp). **c)** The percent of predicted proteins for which annotation information could be found using eggNOG mapper (resulting annotations consist of KEGG KOs (KEGG orthology identifiers), PFAM (protein families), GO (Gene Ontology categories), EC (Enzyme Commission numbers), KEGG Pathway identifiers, KEGG Module descriptions, eggNOG taxonomic classification, COG (clusters of orthologous groups)) and MMseqs2 alignment to both a custom extended version of PhyloDB and EukProt, both with a 60% and 90% sequence identity cut-off threshold value. **d)** Venn diagram showing the number of predicted proteins with a PhyloDB taxonomic annotation (>60% sequence identity with reference), and/or a EukProt taxonomic annotation

(>60% sequence identity with reference), and/or functionally annotated predicted proteins with eggNOG functional information.

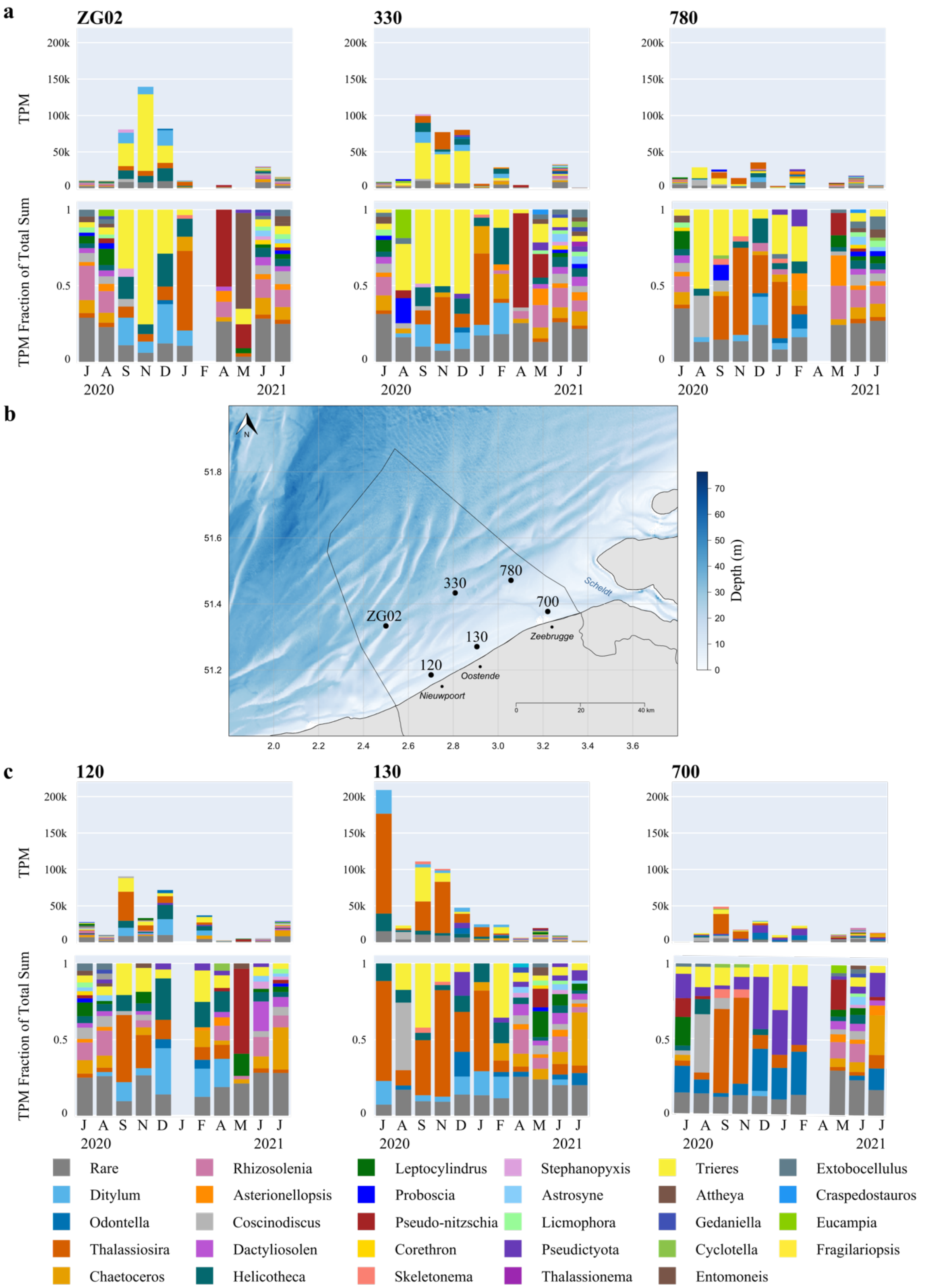

Supplementary Figure 2. Monthly relative abundances of EukProt diatom genera at each sampling station. a) Monthly relative transcript abundance and relative abundance fraction of diatom genera annotated using EukProt (>90% sequence identity), per offshore sampling

station. The relative transcript abundance of a diatom genus represents the sum of TPM of transcripts annotated to that genus. The relative abundance fraction of a genus in a given sample was calculated as the sum of TPM for that genus for a given sample, divided by the total TPM of all diatom genera found in that sample, ignoring transcripts not annotated to specific diatom genera. When the relative abundance of a genus was <2 %, it was labelled as 'rare'. **b)** Spatial location of the 6 sampling stations in the Belgian North Sea. **c)** Monthly relative transcript abundance and relative abundance fraction of diatom genera annotated using EukProt, per nearshore sampling station. Relative abundances and fractions were calculated as for the offshore stations.

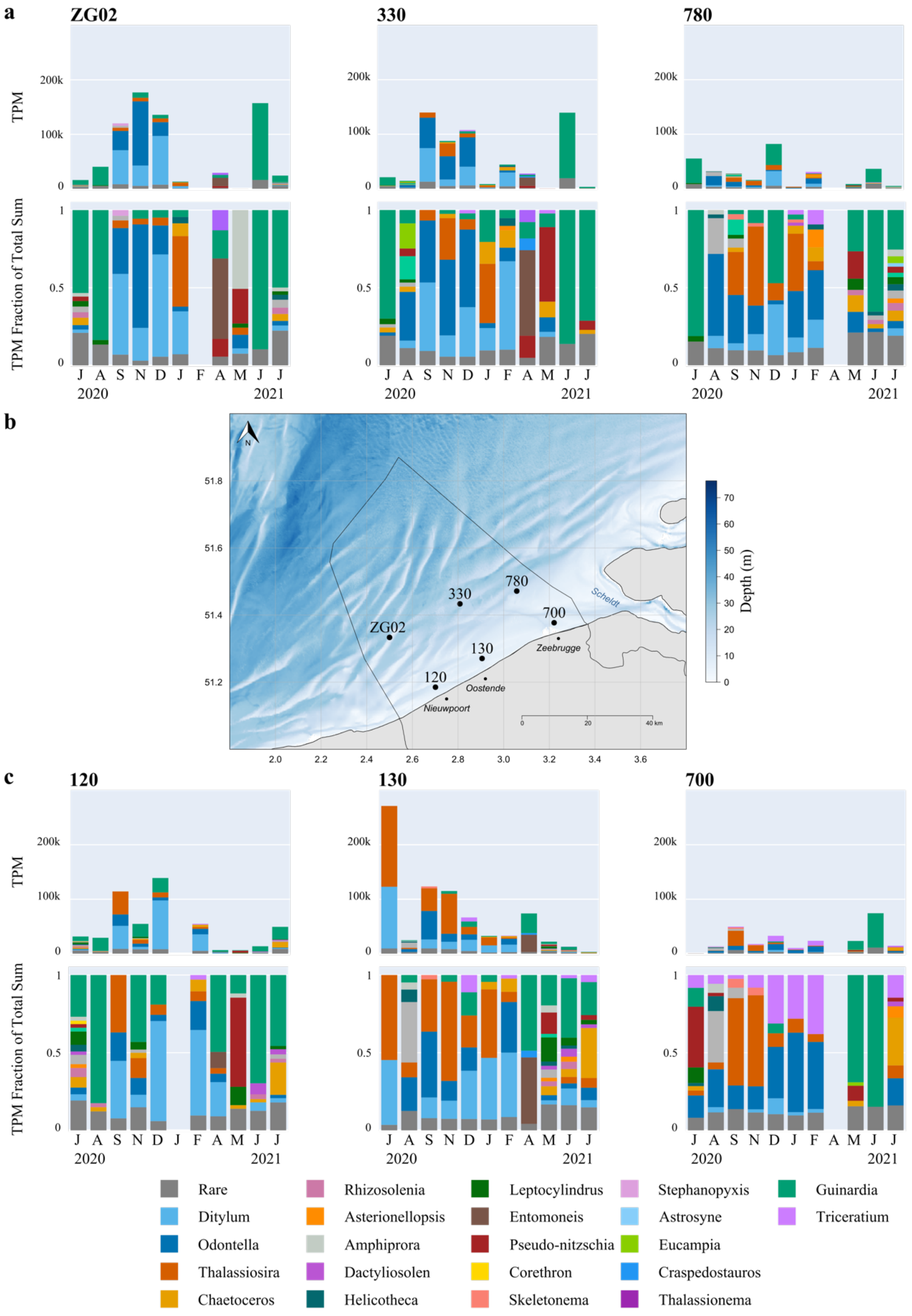

Supplementary Figure 3. Monthly relative abundances of PhyloDB diatom genera at each sampling station. a) Monthly relative transcript abundance and relative abundance fraction of diatom genera annotated using PhyloDB (>90% sequence identity), per offshore sampling

station. The relative transcript abundance of a diatom genus represents the sum of TPM of transcripts annotated to that genus. The relative abundance fraction of a genus in a given sample was calculated as the sum of TPM for that genus, divided by the total TPM of all diatom genera found in that sample, ignoring transcripts not annotated to specific diatom genera. When the relative abundance of a genus was <2 %, it was labelled as 'rare'. **b)** Spatial location of the 6 sampling stations in the Belgian North Sea. **c)** Monthly relative transcript abundance and relative abundance fraction of diatom genera annotated using PhyloDB, per nearshore sampling station. Relative transcript abundances and fractions were calculated as for the offshore stations.

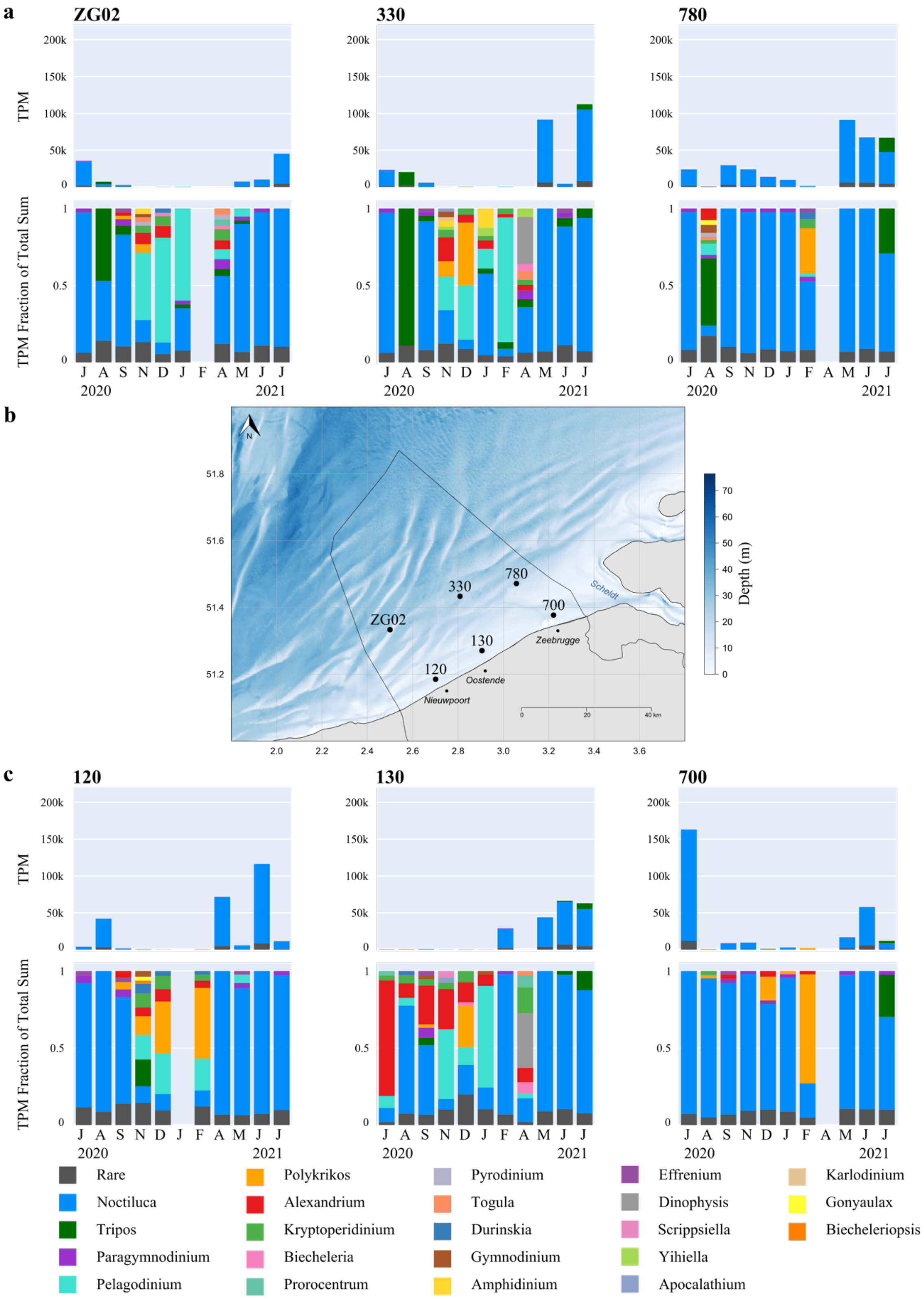

Supplementary Figure 4. Monthly relative abundances of EukProt dinoflagellate genera at each station. a) Monthly relative transcript abundance and relative abundance fraction of dinoflagellate genera annotated using EukProt (>90% sequence identity), per offshore

sampling station. The relative transcript abundance of a dinoflagellate genus represents the sum of TPM of transcripts annotated to that genus. The relative abundance fraction of a genus in a given sample was calculated as the sum of TPM for that genus, divided by the total TPM of all dinoflagellate genera found in that sample, ignoring transcripts not annotated to specific dinoflagellate genera. When the relative abundance of a genus was <2 %, it was labelled as ‘rare’. **b)** Spatial location of the 6 sampling stations in the Belgian Part of the North Sea. **c)** Monthly relative transcript abundance and relative abundance fraction of dinoflagellate genera annotated using EukProt, per nearshore sampling station. Relative abundances and fractions were calculated as for the offshore stations.

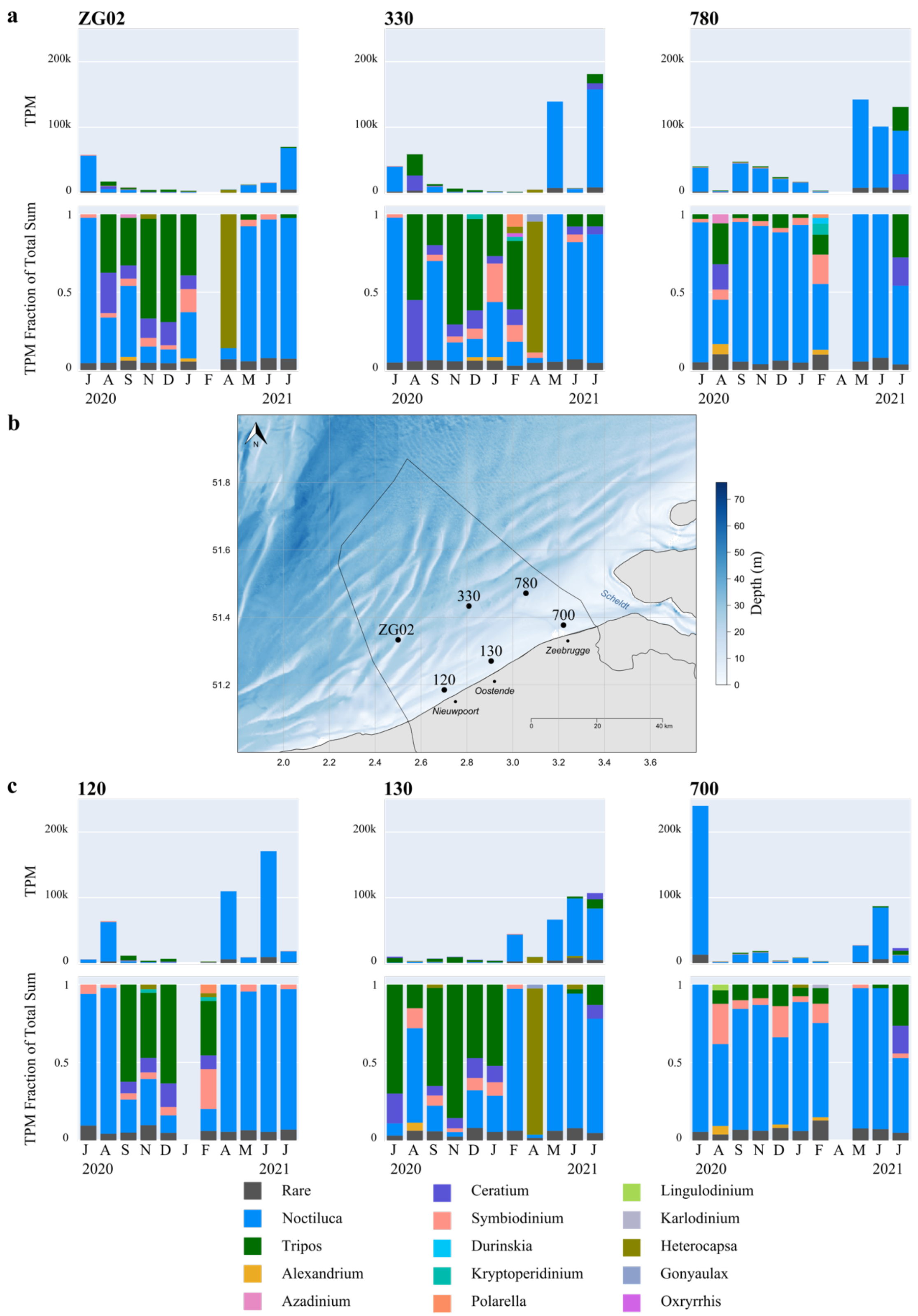

Supplementary Figure 5. Monthly relative abundances of PhyloDB dinoflagellate genera at each station. a) Monthly relative transcript abundance and relative abundance fraction of dinoflagellate genera annotated using PhyloDB (>90% sequence identity), per offshore

sampling station. The relative transcript abundance of a dinoflagellate genus represents the sum of TPM of transcripts annotated to that genus. The relative abundance fraction of a genus in a given sample was calculated as the sum of TPM for that genus, divided by the total TPM of all dinoflagellate genera found in that sample. When the relative abundance of a genus was <2 %, it was labelled as 'rare'. **b)** Spatial location of the 6 sampling stations in the Belgian Part of the North Sea. **c)** Monthly relative transcript abundance and relative abundance fraction of dinoflagellate genera annotated using PhyloDB, per nearshore sampling station. Relative transcript abundances and fractions were calculated as for the offshore stations.

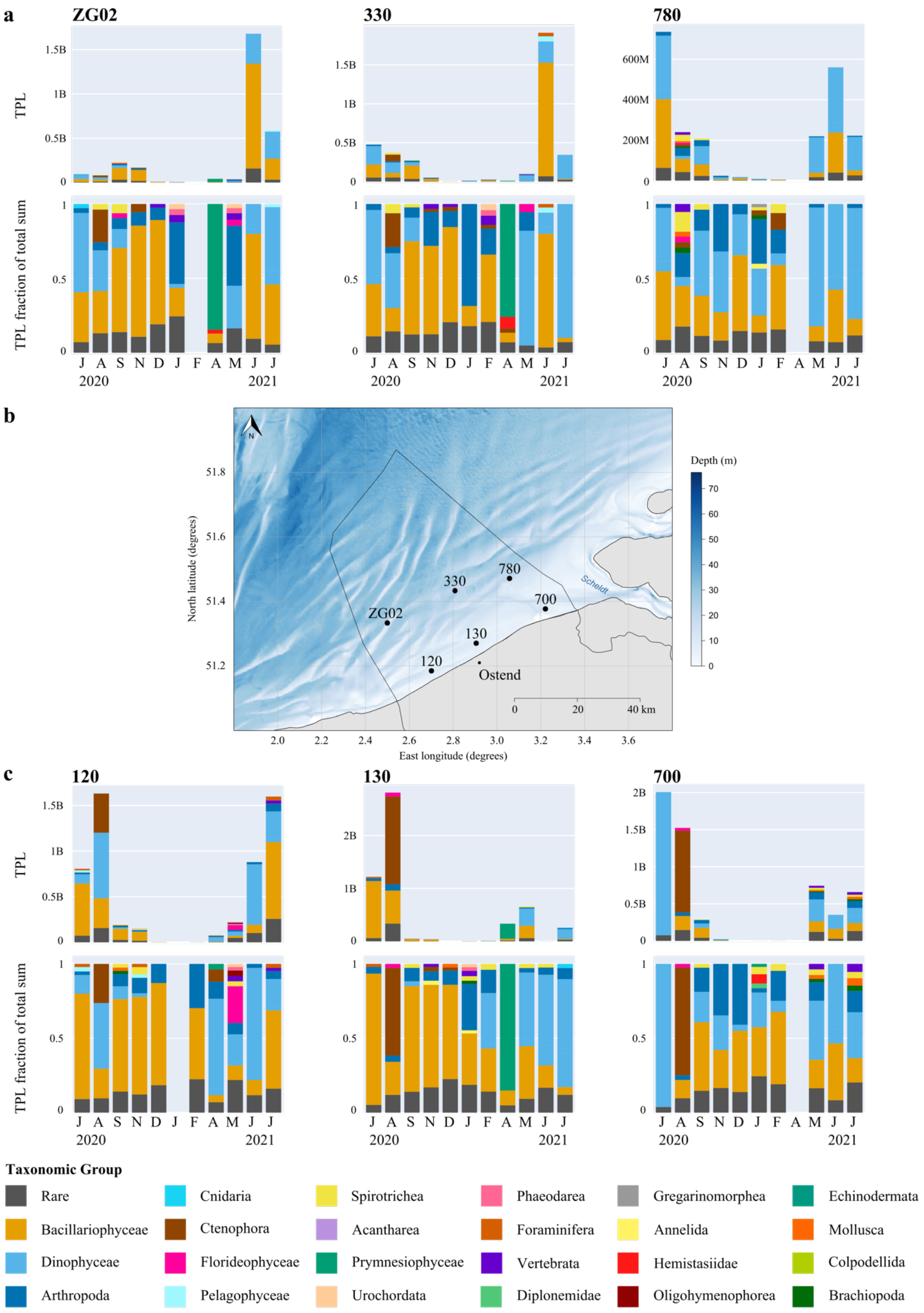
a
ZG02
330
780
TPL
TPL fraction of total sum
J A S N D J F A M J J
2020
2021
b
North latitude (degrees)
East longitude (degrees)
Depth (m)
ZG02
330
780
700
130
120
Ostend
Scheldt
0 20 40 km
c
120
130
700
Taxonomic Group
Rare
Bacillariophyceae
Dinophyceae
Arthropoda
Cnidaria
Ctenophora
Florideophyceae
Pelagophyceae
Spirotrichea
Acantharea
Prymnesiophyceae
Urochordata
Phaeodarea
Foraminifera
Vertebrata
Diplonemidae
Gregarinomorphea
Annelida
Hemistasiidae
Oligohymenophorea
Echinodermata
Mollusca
Colpodellida
Brachiopoda

Supplementary Figure 6. Monthly turnover in estimated transcripts per L and relative taxonomic composition at each sampling station. a) Monthly estimated transcripts per liter (TPL, top panels) and TPL fractions (bottom panels) for taxonomic groups annotated using EukProt (>60% sequence identity), per offshore sampling station. TPL were calculated based on the recovery of ERCC92 transcript standards and processed volumes during RNA extraction and library preparation (see Supplementary Methods). TPL fractions were calculated as the sum of TPL for a group for a given sample, divided by the total TPL of all groups found in that sample, excluding unannotated transcripts. When the TPL fraction of a group was <2 %, it was labelled as 'rare'. **b)** Spatial location of the 6 sampling stations in the Belgian North Sea. **c)** Monthly estimated TPL (top panels) and TPL fraction (bottom panels) for taxonomic groups annotated using EukProt (>60% sequence identity), per nearshore sampling station. TPL and TPL fractions were calculated as for the offshore stations. Small differences between the relative abundance fractions displayed in Fig. 3 and the TPL fractions displayed here are due to removal of transcripts with expression < 1 TPM in Fig. 3 versus removal of transcripts with expression < 1 TPL here.

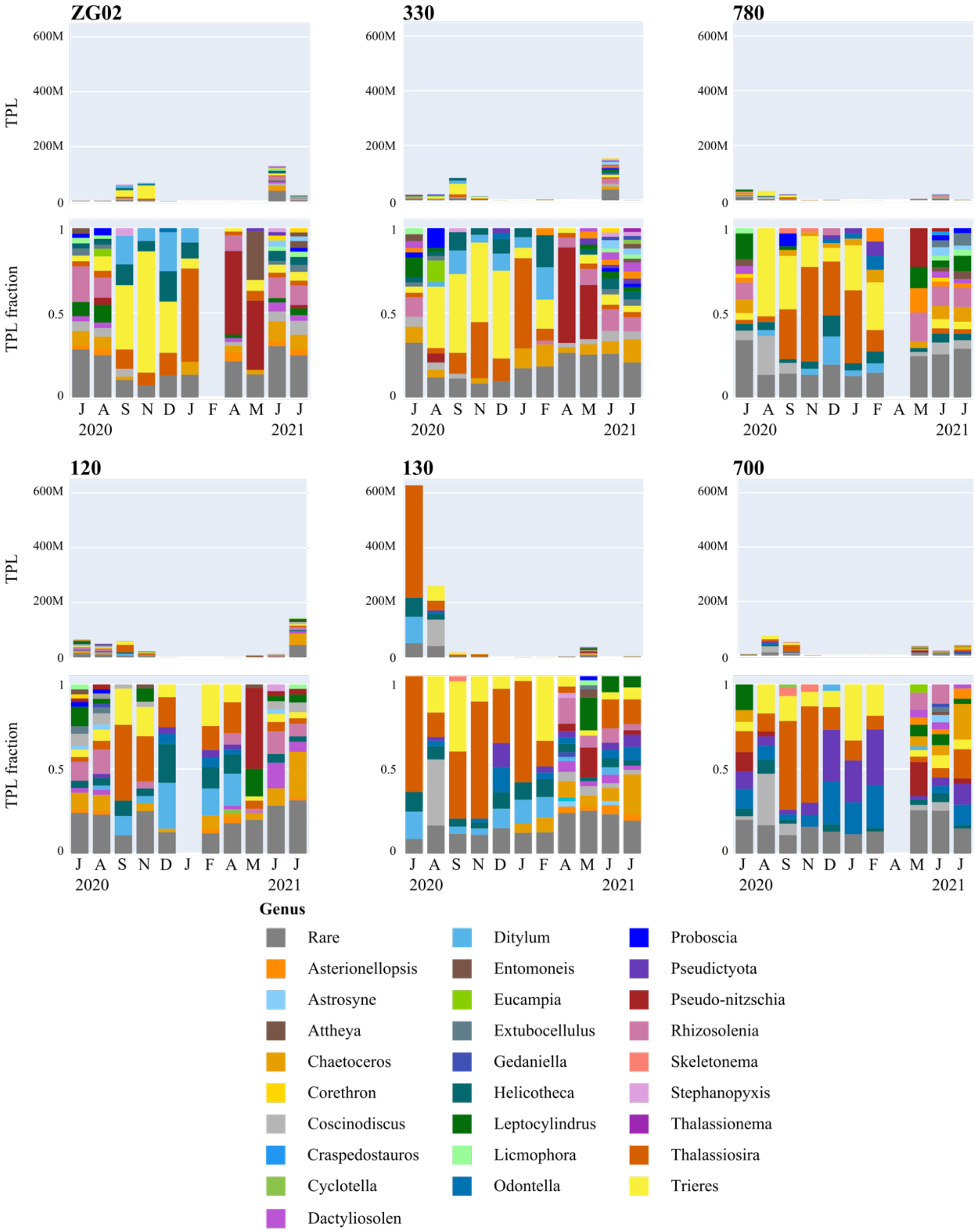

Supplementary Figure 7. Monthly turnover in estimated diatom transcripts per L and relative taxonomic composition at each sampling station. Monthly estimated transcripts per liter (TPL, top panels) and TPL fractions (bottom panels) for diatom genera annotated using EukProt (>90% sequence identity), per sampling station. TPL were calculated based on the recovery of ERCC92 transcript standards and processed volumes during RNA extraction and library preparation (see Supplementary Methods). TPL fractions were calculated as the sum of TPL for a group in a given sample, divided by the total TPL of all groups found in that sample, ignoring transcripts not annotated to specific diatom genera. When relative TPL fraction of a group was <2 %, it was labelled as 'rare'.

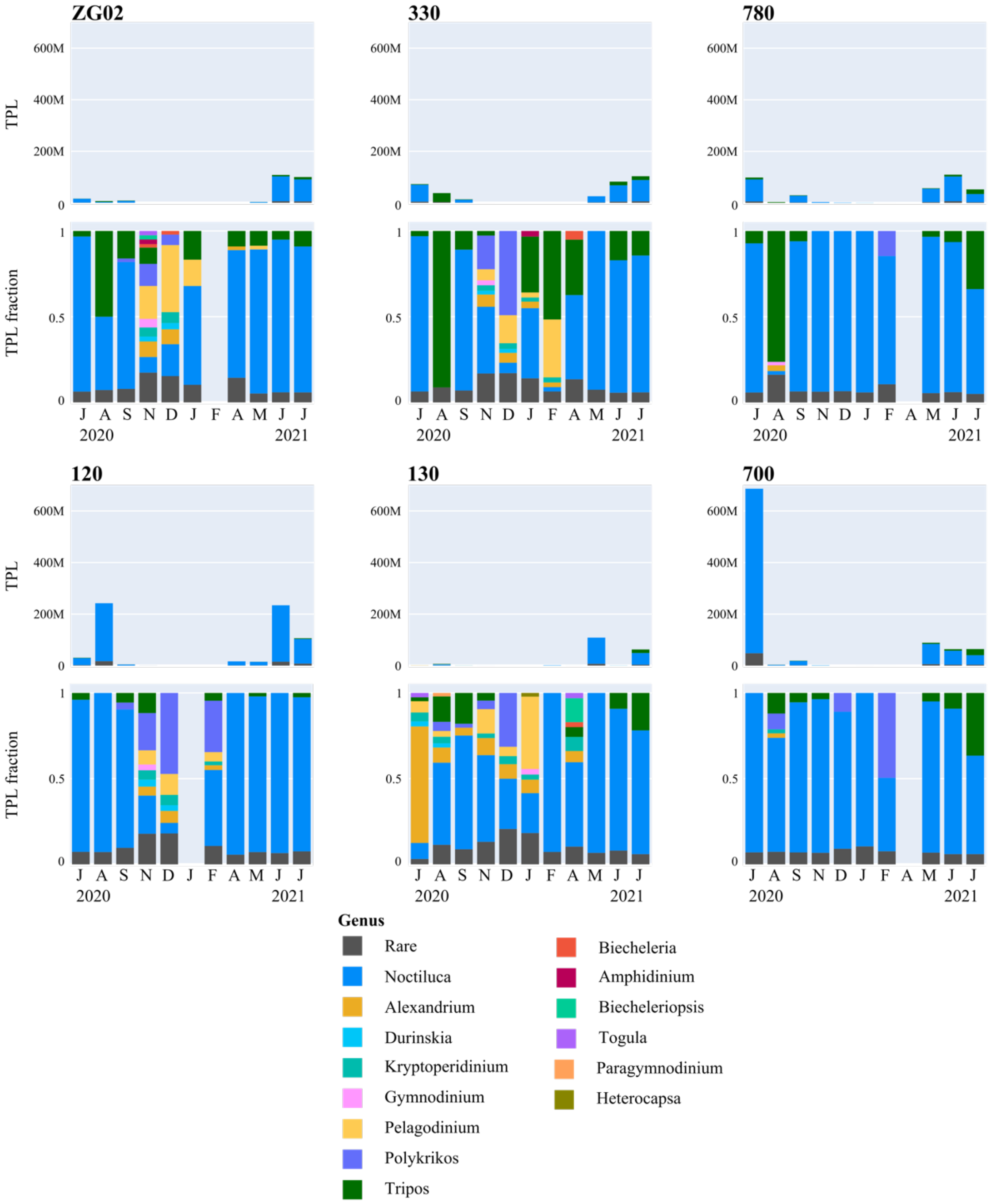

Supplementary Figure 8. Monthly turnover in estimated dinoflagellate transcripts per L and relative taxonomic composition at each sampling station. Monthly estimated transcripts per liter (TPL, top panels) and TPL fractions (bottom panels) for dinoflagellate genera annotated using EukProt (>90% sequence identity), per sampling station. TPL were calculated based on the recovery of ERCC92 transcript standards and processed volumes during RNA extraction and library preparation (see Supplementary Methods). TPL fractions were calculated as the sum of TPL for a group in a given sample, divided by the total TPL of all groups found in that sample, ignoring transcripts not annotated to specific dinoflagellate genera. When relative TPL fraction of a group was <2 %, it was labelled as 'rare'.

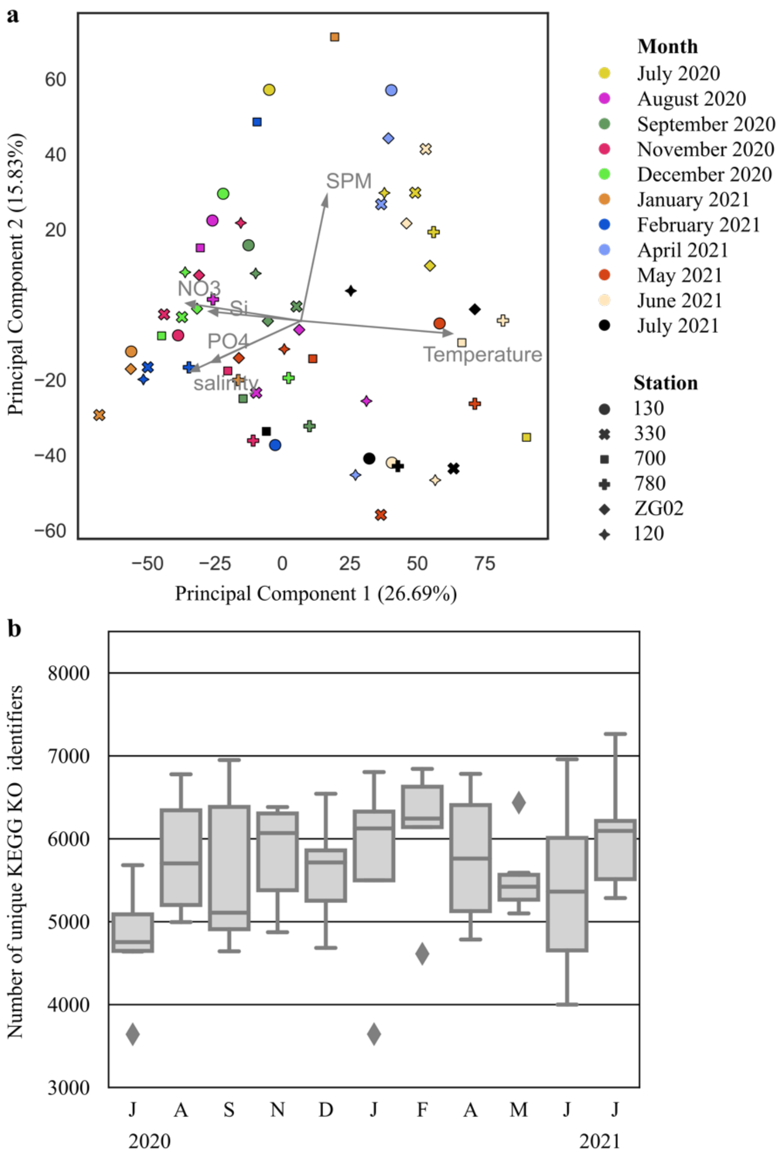

Supplementary Figure 9. Seasonal changes in ecosystem functional richness and composition. a) Principal Component Analysis of log transformed TPM expression data (summed per KO identifier per sample). Colours represent sampling months; shapes reflect sampling stations. Arrows indicate correlation of principal components with environmental parameters. **b)** Boxplot showing the number of unique KO identifiers detected per month per sample from July 2020 to July 2021. Boxes encompass the interquartile range, with the central line showing the median. Whiskers extend to the furthest data points within 1.5 times the interquartile range below the first or above the third quartile.

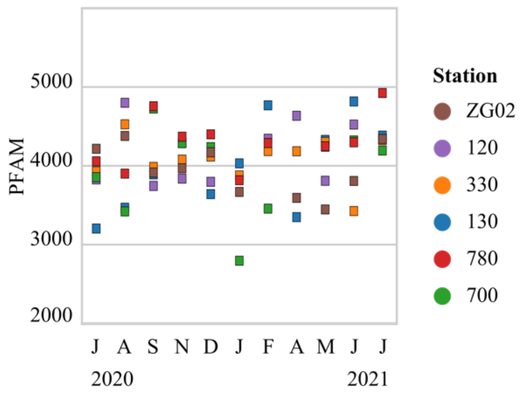

Supplementary Figure 10. The number of unique PFAM families observed per sample per month, coloured according to sampling station.

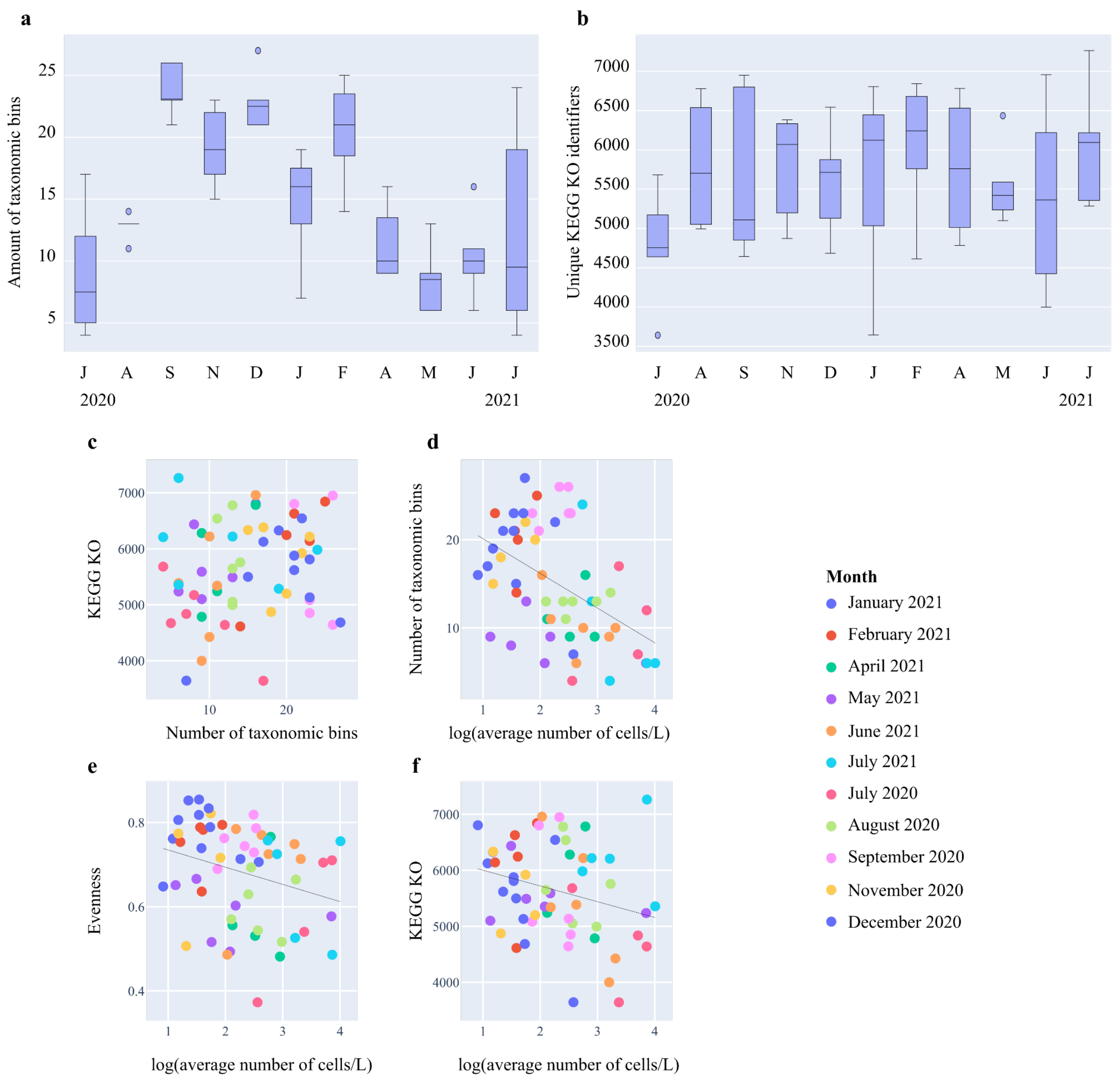

Supplementary Figure 11. The relation between functional richness, species richness, and biomass. a-b) Boxplots of the number of active species (taxonomic species bins with a minimum of 100 transcripts with non-zero expression in at least one sample), and the number of unique KEGG KO identifiers in a sample over time. Boxes encompass interquartile range, with the central line showing median. Whiskers extend to the furthest data point within 1.5 times the interquartile range. **c)** The number of active species in relation to the number of unique KEGG identifiers across samples ($r(60) = 0.18$, $p = 0.151$). **d)** The log-transformed average estimate of cells per L of sea water in relation to the number of active species ($r(55) = -0.48$, $p = 0.0002$). **e)** The log-transformed average number of cells per L in relation to the evenness (Shannon diversity divided by the number of species) ($r(55) = -0.28$, $p = 0.0364$). **f)**

The log-transformed average number of cells per L in relation to the number of unique KEGG identifiers across samples (r(55) = 0.26, p = 0.0492).

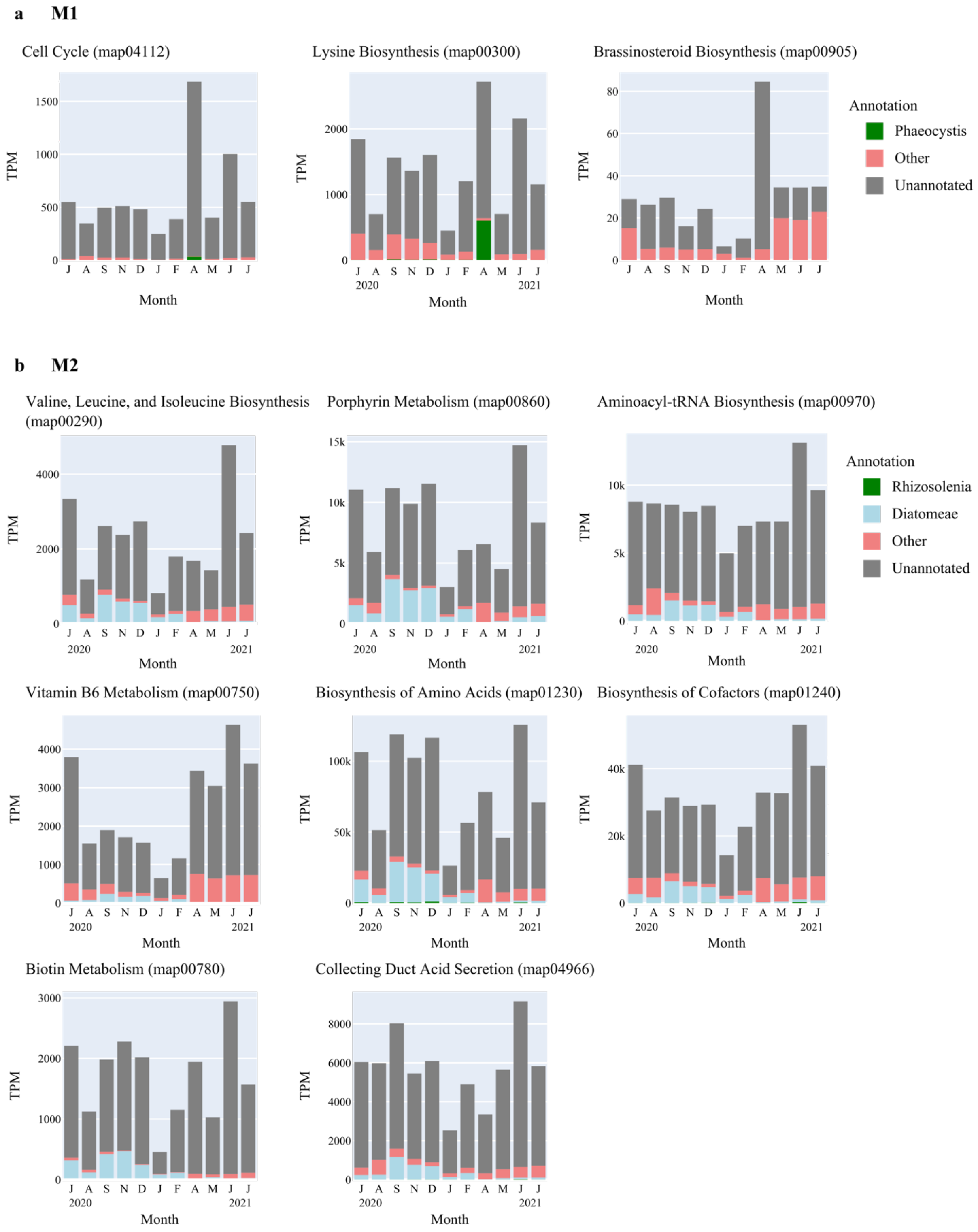

Supplementary Figure 12. Expression of pathways characteristic of module 1 and 2. Each plot represents a characteristic pathway for module 1 (panel a) or module 2 (panel b), as determined by Mann-Whitney U tests assessing the correlation between the TPM expression levels of a pathways' KO identifiers and the eigengenes of the respective module. Each plot shows the monthly TPM values for a characteristic pathway, incorporating all transcripts annotated to KOs involved in this pathway and summed across stations. Green bars indicate the fraction of transcript expression annotated to the genus whose absolute abundance profile is most highly correlated with the eigengene expression of the module concerned. Blue bars indicate the fraction of transcript expression annotated to the same taxonomic class as the genus represented by the green bars. Red bars indicate fraction of transcript expression annotated to other groups. Grey bars denote taxonomically unannotated transcripts.

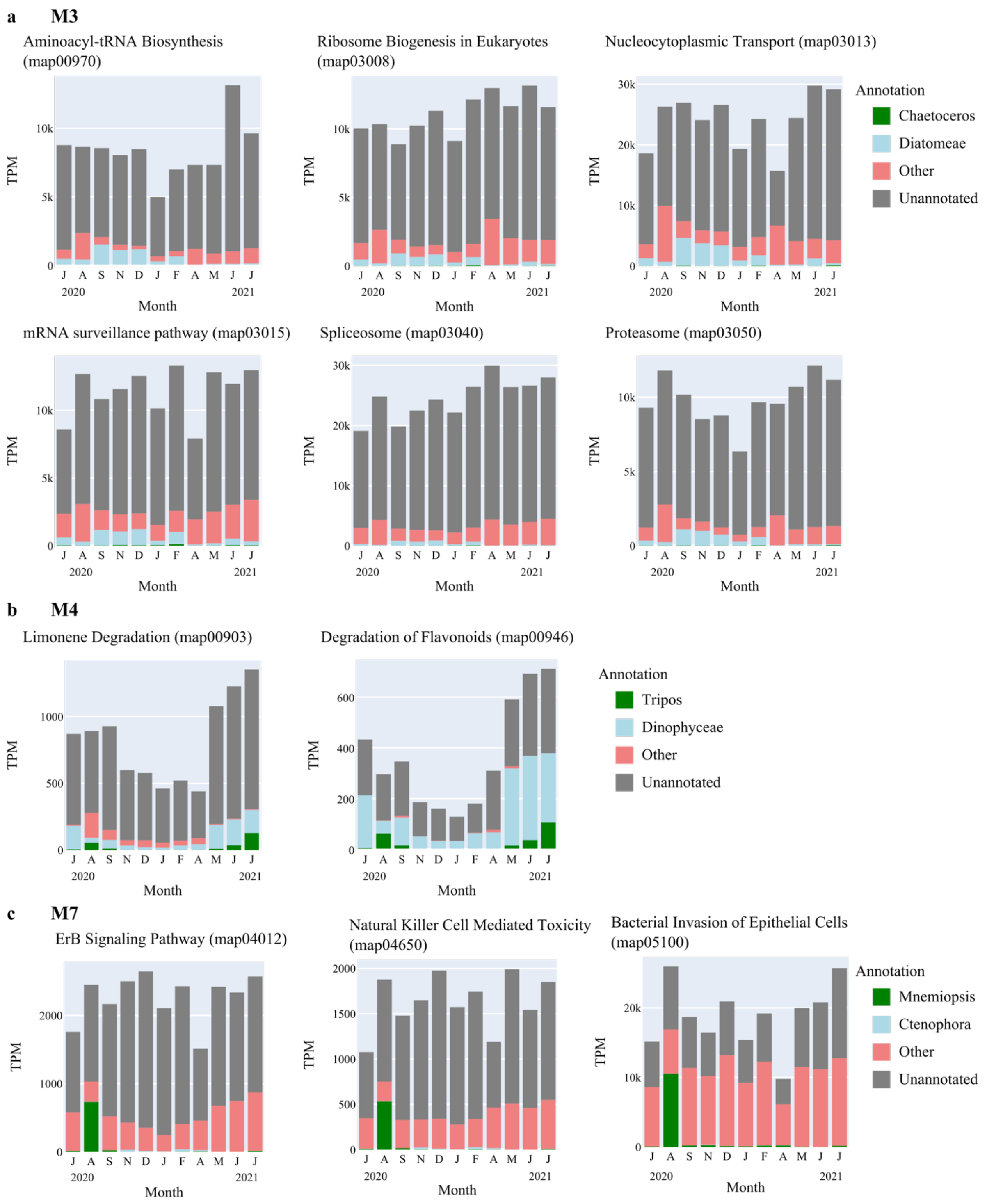

Supplementary Figure 13. Expression of pathways characteristic of module 3, 4, and 7. Each plot represents a characteristic pathway for module 3 (panel a), module 4 (panel b) or module 7 (panel c) as determined by Mann-Whitney U tests assessing the correlation between the TPM expression levels of a pathways' KO identifiers and the eigengenes of the respective module. Each plot shows the monthly TPM values for a characteristic pathway, incorporating all transcripts annotated to KOs involved in this pathway and summed across stations. Green

bars indicate the fraction of transcript expression annotated to the genus whose absolute abundance profile is most highly correlated with the eigengene expression of the module concerned. Blue bars indicate the fraction of transcript expression annotated to the same taxonomic class as the genus represented by the green bars. Red bars indicate fraction of transcript expression annotated to other groups. Grey bars denote taxonomically unannotated transcripts.

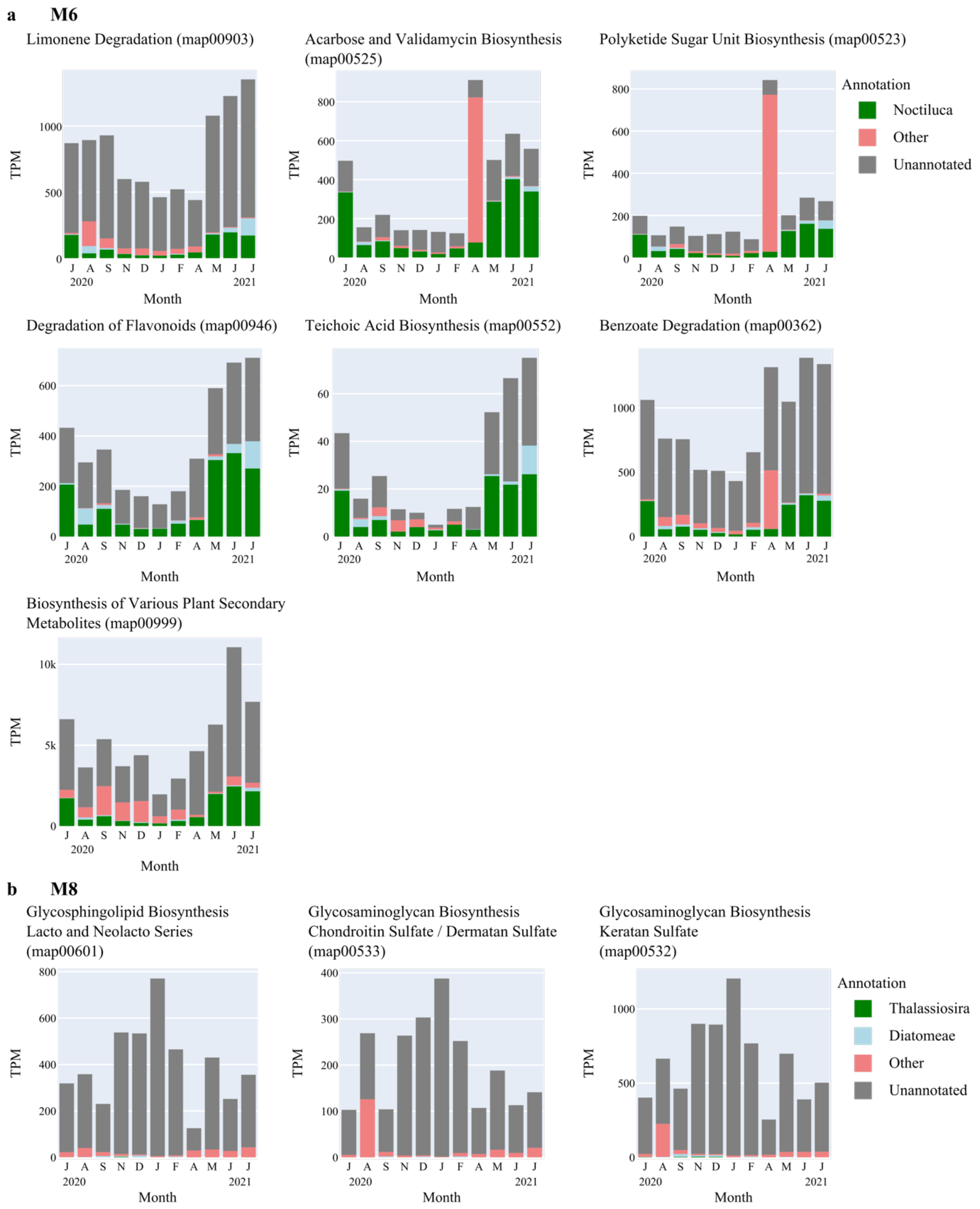

Supplementary Figure 14. Expression of pathways characteristic of module 6 and 8. Each plot represents a characteristic pathway for module 6 (panel a) or module 8 (panel b), as determined by Mann-Whitney U tests assessing the correlation between the TPM expression levels of a pathways' KO identifiers and the eigengenes of the respective module. Each plot shows the monthly TPM values for a characteristic pathway, incorporating all transcripts annotated to KOs involved in this pathway and summed across stations. Green bars indicate

the fraction of transcript expression annotated to the genus whose absolute abundance profile is most highly correlated with the eigengene expression of the module concerned. Blue bars indicate the fraction of transcript expression annotated to the same taxonomic class as the genus represented by the green bars. Red bars indicate fraction of transcript expression annotated to other groups. Grey bars denote taxonomically unannotated transcripts.

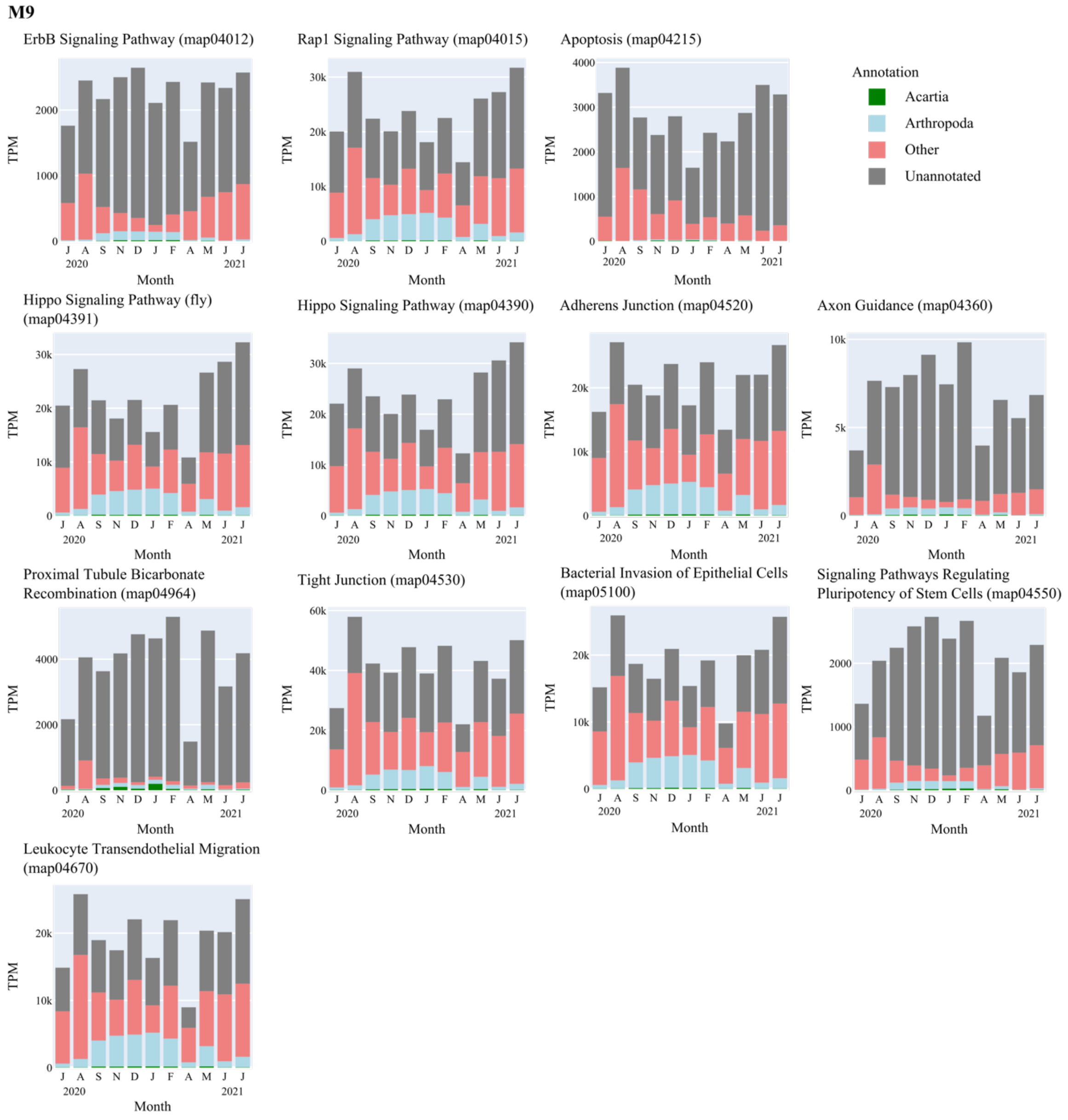

Supplementary Figure 15. Expression of pathways characteristic of module 9. Characteristic pathways for module 9, as determined by Mann-Whitney U tests assessing the correlation between the TPM expression levels of a pathways' KO identifiers and the eigengenes of the respective module. Each plot shows the monthly TPM values for a

characteristic pathway, incorporating all transcripts annotated to KOs involved in this pathway and summed across stations. Green bars indicate the fraction of transcript expression annotated to the genus whose absolute abundance profile is most highly correlated with the eigengene expression of the module concerned. Blue bars indicate the fraction of transcript expression annotated to the same taxonomic class as the genus represented by the green bars. Red bars indicate fraction of transcript expression annotated to other groups. Grey bars denote taxonomically unannotated transcripts.

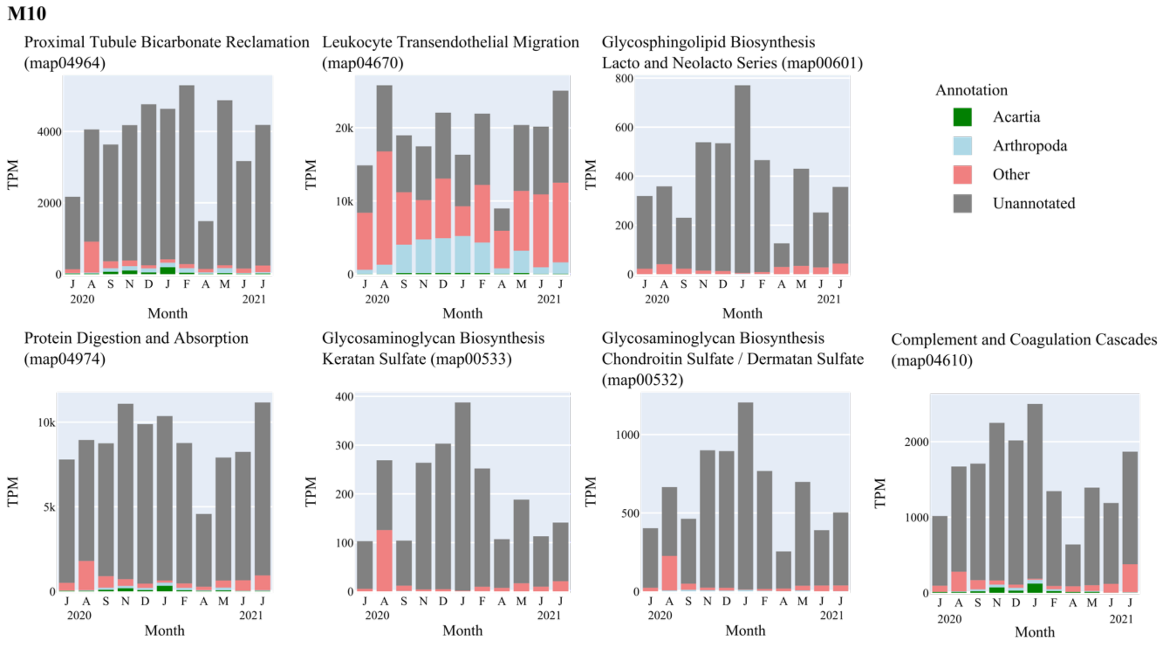

Supplementary Figure 16. Expression of pathways characteristic of module 10. Characteristic pathways for module 10, as determined by Mann-Whitney U tests assessing the correlation between the TPM expression levels of a pathways' KO identifiers and the eigengenes of the respective module. Each plot shows the monthly TPM values for a characteristic pathway, incorporating all transcripts annotated to KOs involved in this pathway and summed across stations. Green bars indicate the fraction of transcript expression annotated to the genus whose absolute abundance profile is most highly correlated with the eigengene expression of the module concerned. Blue bars indicate the fraction of transcript expression annotated to the same taxonomic class as the genus represented by the green bars. Red bars indicate fraction of transcript expression annotated to other groups. Grey bars denote taxonomically unannotated transcripts.

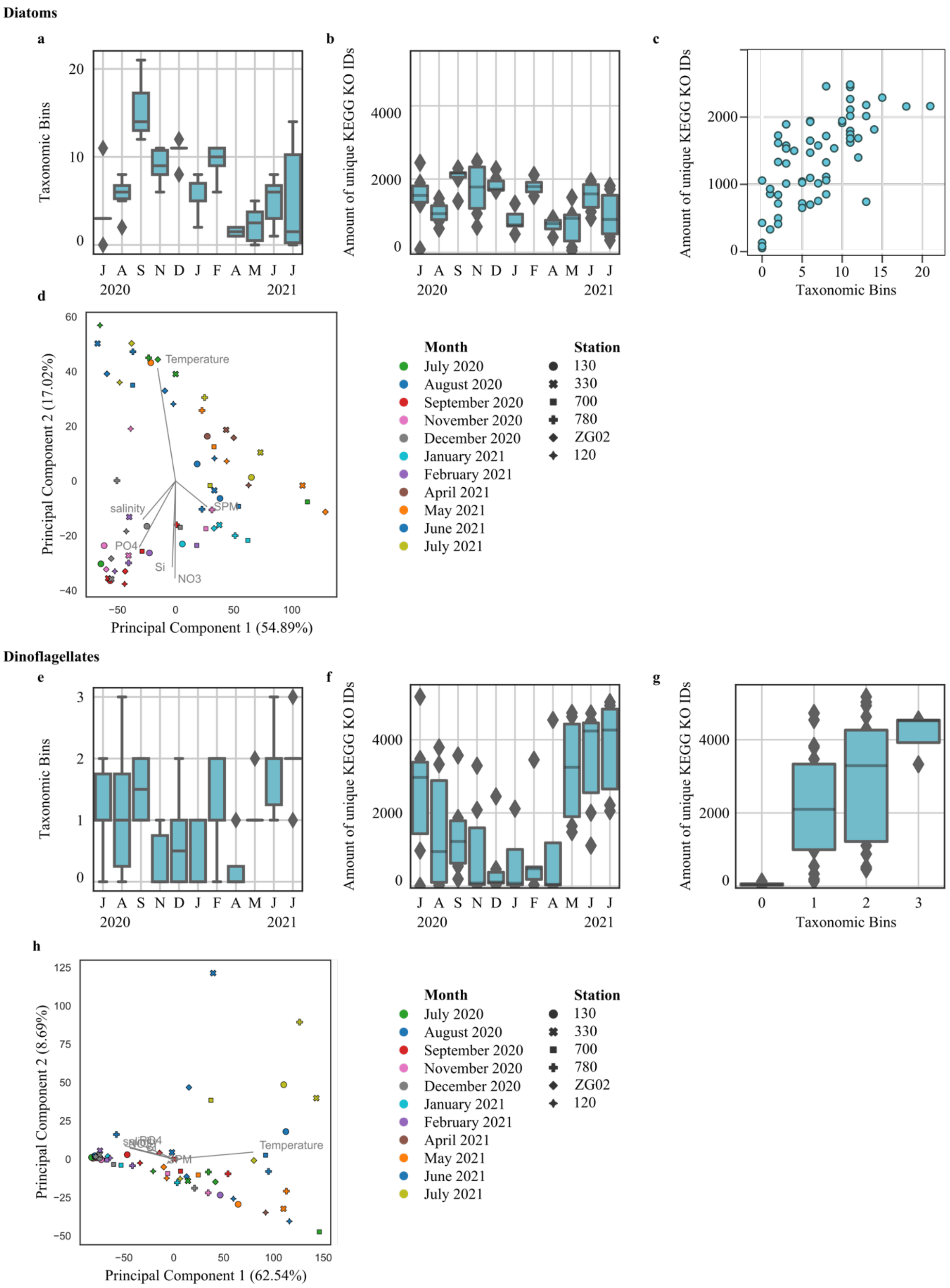

Supplementary Figure 17. The occurrence of taxonomic bins, functional richness and functional diversity for diatoms and dinoflagellates annotated using EukProt. a & e) Boxplots of the number of active diatom and dinoflagellate species (taxonomic species bins

with a minimum of 100 transcripts with non-zero expression in at least one sample) in a sample over time. Boxes encompass interquartile range, with the central line showing median. Diamonds represent outliers. **b & f)** Boxenplots of the number of unique KEGG KO IDs in a sample over time. Boxes encompass interquartile range, with the central line showing median. Diamonds represent outliers. **c)** Scatterplot depicting the relation between the amount of unique KEGG KO IDs and the number of active species. The grey line traces a fitted exponential model. **g)** Boxenplot of the number of unique KEGG KO IDs in relation to the number of active species. Boxes encompass interquartile range, with the central line showing median. Diamonds represent outliers. **d-h)** Principal Component Analyses on the log-transformed KEGG KO ID expression data from diatom and dinoflagellate EukProt annotated transcripts.

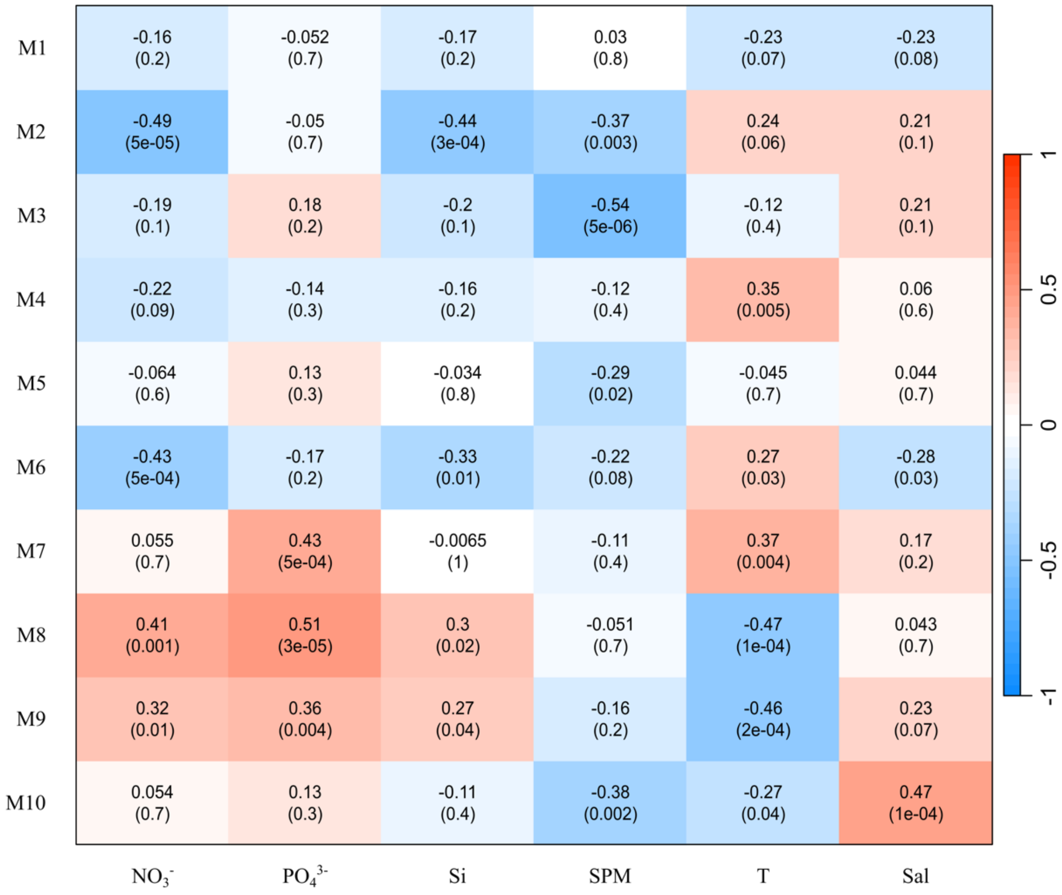

Supplementary Figure 18. Correlation between module eigengene expression and environmental parameters. Numbers indicate Pearson's correlations and their significance.

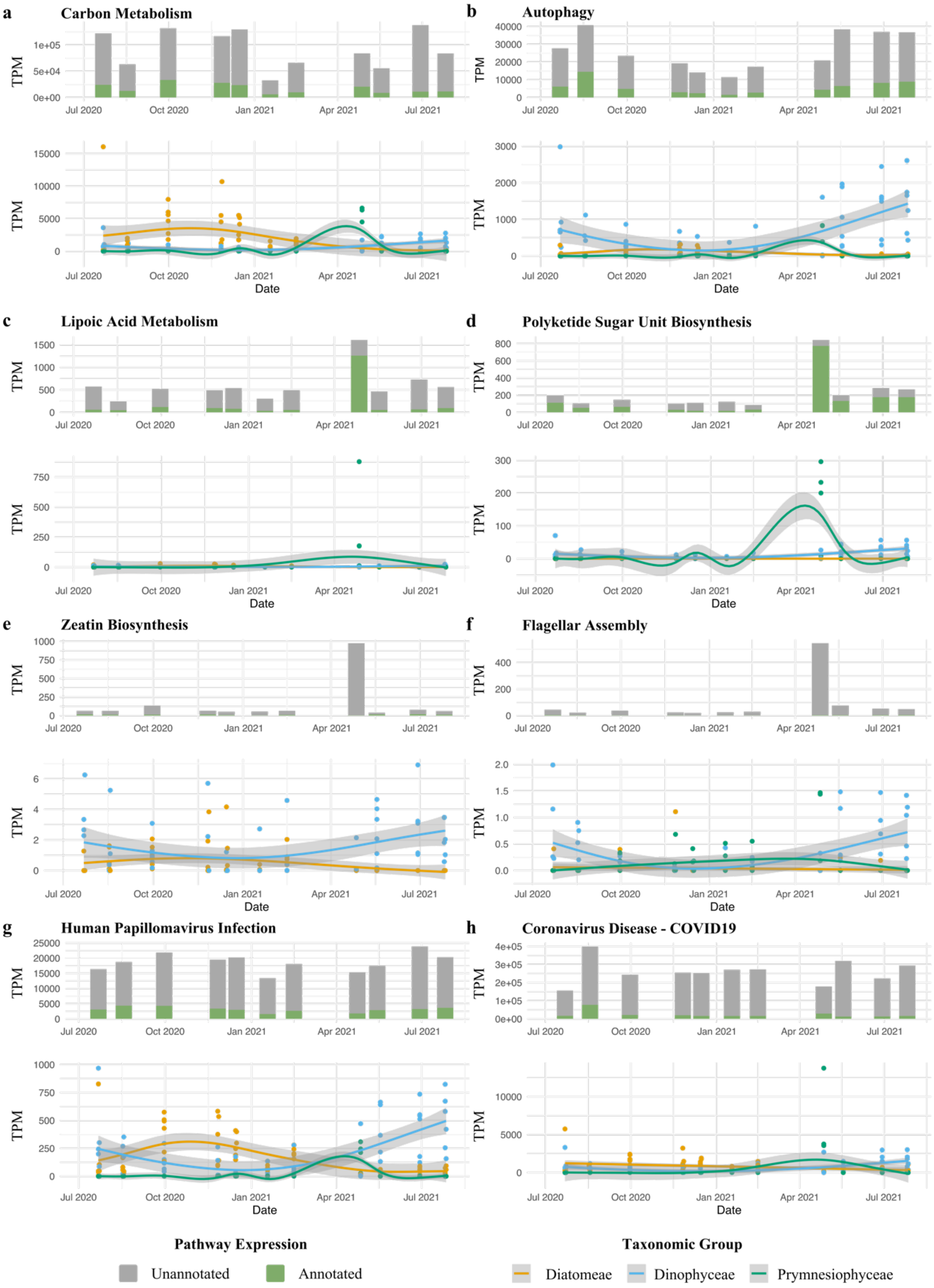

Supplementary Figure 19. Expression of selected pathways and GAM regression fits for the expression of diatom, dinoflagellate, or *Phaeocystis* transcripts. Each panel shows the TPM expression profiles of transcripts associated with a given KEGG pathway of interest (top

figures), alongside Generalised Additive Model (GAM) regression fits to the summed TPM expression profiles of pathway transcripts that were annotated to diatoms, dinoflagellates, or *Phaeocystis* (>90% sequence identity) (bottom figures). The top figure in each panel contrasts the summed TPM expression levels of taxonomically annotated versus unannotated transcripts associated with the KEGG pathway concerned. The lines are GAM fits, with the shaded areas representing the 95% confidence intervals for the smoothed mean estimates. 8 pathways are displayed: **(a)** carbon metabolism, **(b)** autophagy, **(c)** lipoic acid metabolism, **(d)** polyketide sugar unit biosynthesis, **(e)** zeatin biosynthesis, **(f)** flagellar assembly, **(g)** human papillomavirus infection, and **(h)** coronavirus disease.

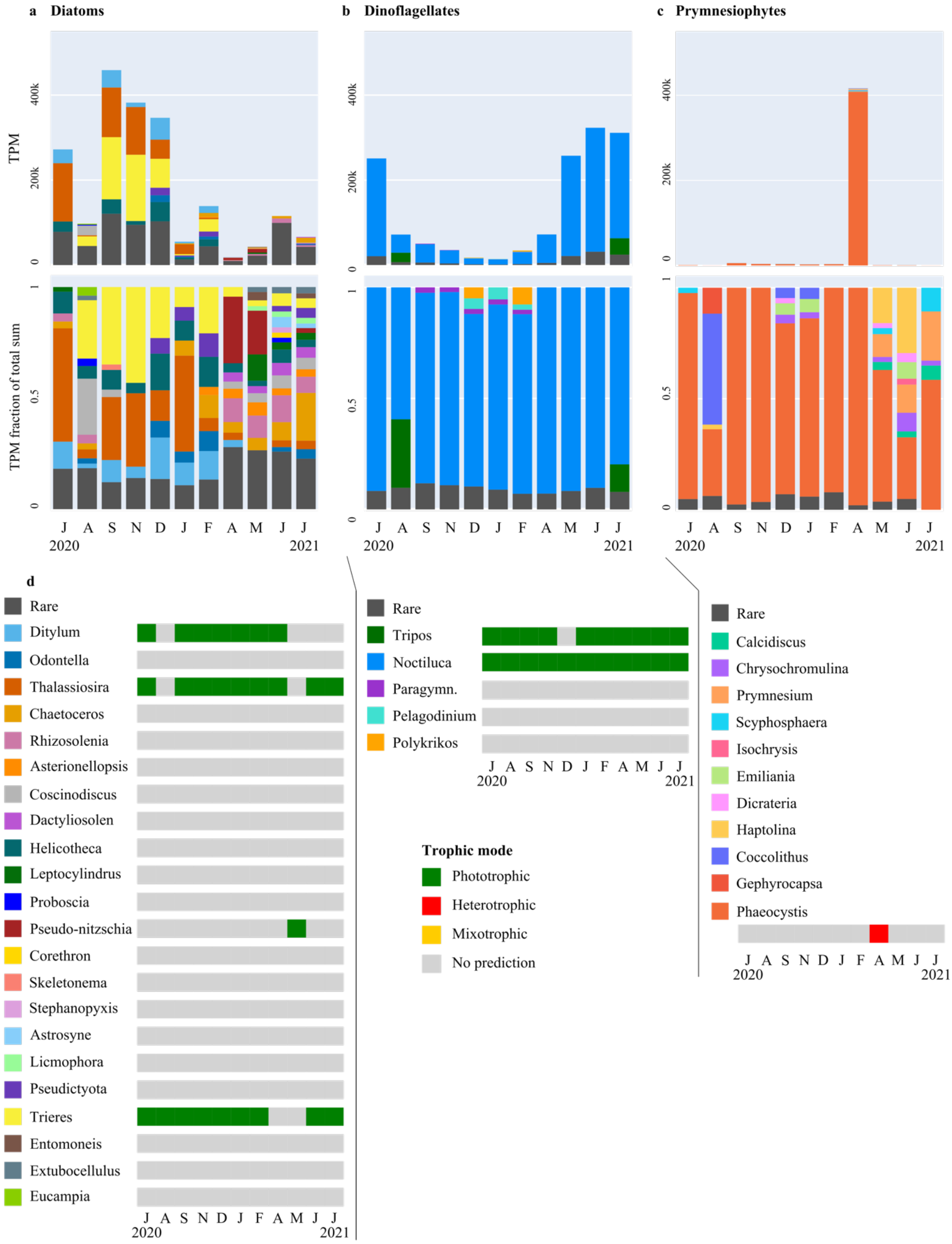

Supplementary Figure 20. Relative turnover in diatom, dinoflagellate, and prymnesiophyte genera and their predicted trophic mode. a) The TPM sum of transcripts annotated to diatom genera from July 2020 to July 2021 (top) and their relative taxonomic

composition across months (bottom). **b)** The TPM sum across sampling stations annotated to dinoflagellate genera from July 2020 to July 2021 (top) and their relative taxonomic composition across months (bottom). **c)** The TPM sum across sampling stations annotated to prymnesiophyte genera from July 2020 to July 2021 (top) and their relative taxonomic composition across months (bottom). **d**) Trophic mode consensus prediction per species per month. Trophic modes could be predicted for taxonomic species bins that contained >800 PFAMs. The monthly consensus prediction was determined by a majority vote across samples (green: phototrophic, red: heterotrophic, grey: no prediction). Taxonomic annotations were obtained using EukProt.

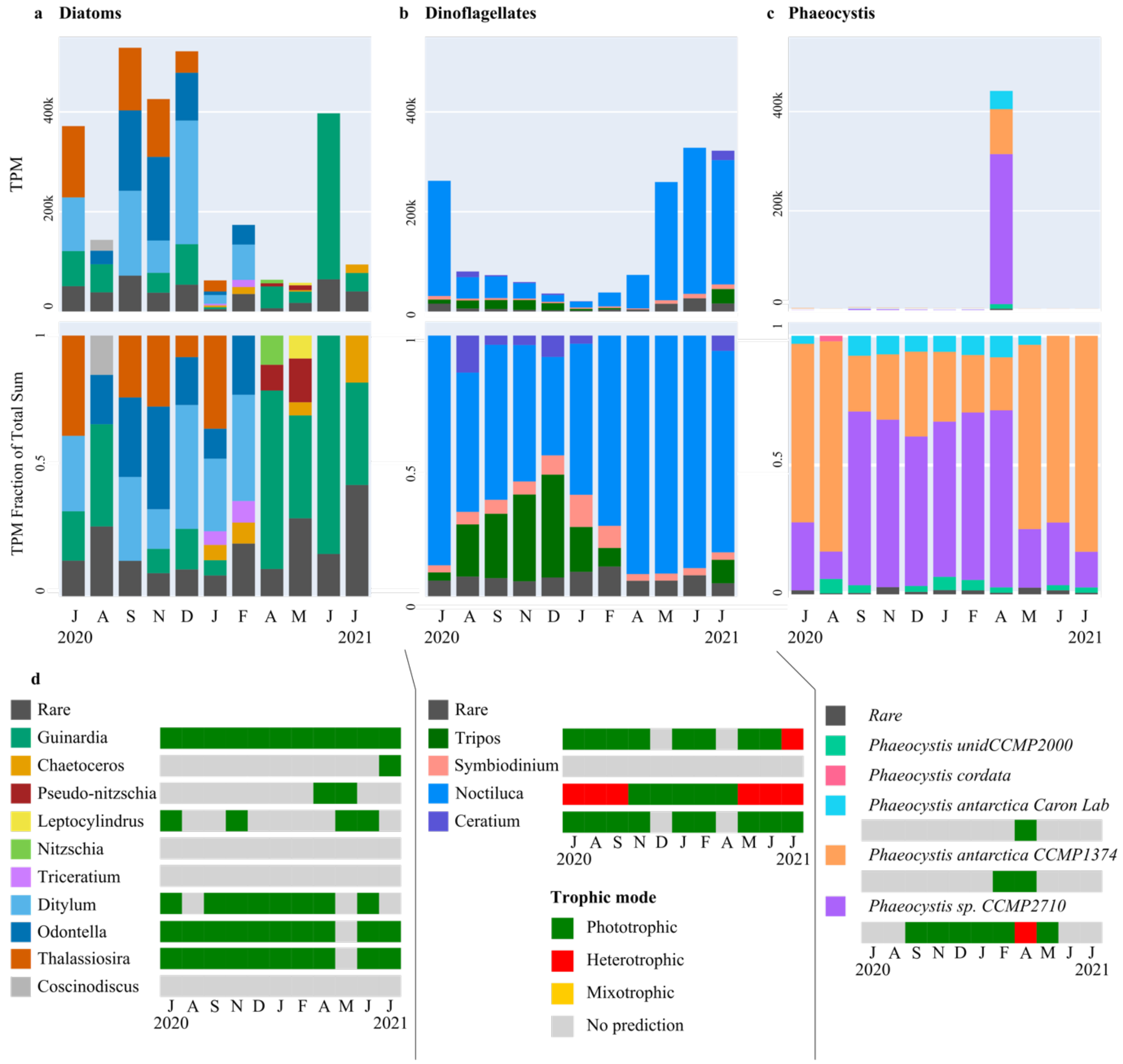

Supplementary Figure 21. Relative turnover in diatom, dinoflagellate, and prymnesiophyte genera and their trophic mode based on PhyloDB taxonomic annotation. **a)** The TPM sum of transcript annotated to diatom genera from July 2020 to July 2021(top) and their relative taxonomic composition across months (bottom). **b)** The TPM sum of transcripts annotated to dinoflagellate genera from July 2020 to July 2021 (top) and their

relative taxonomic composition across months (bottom). **c)** The TPM sum of transcripts annotated to the genus *Phaeocystis* from July 2020 to July 2021 (top) and their relative taxonomic composition across months (bottom). **d**) Trophic mode consensus prediction per month. For taxonomic species bins that contained >800 PFAMs, trophic mode could be predicted per sample. The monthly consensus prediction was determined by a majority vote across samples (green: phototrophic, red: heterotrophic, grey: no prediction). Taxonomic annotations were obtained using PhyloDB.

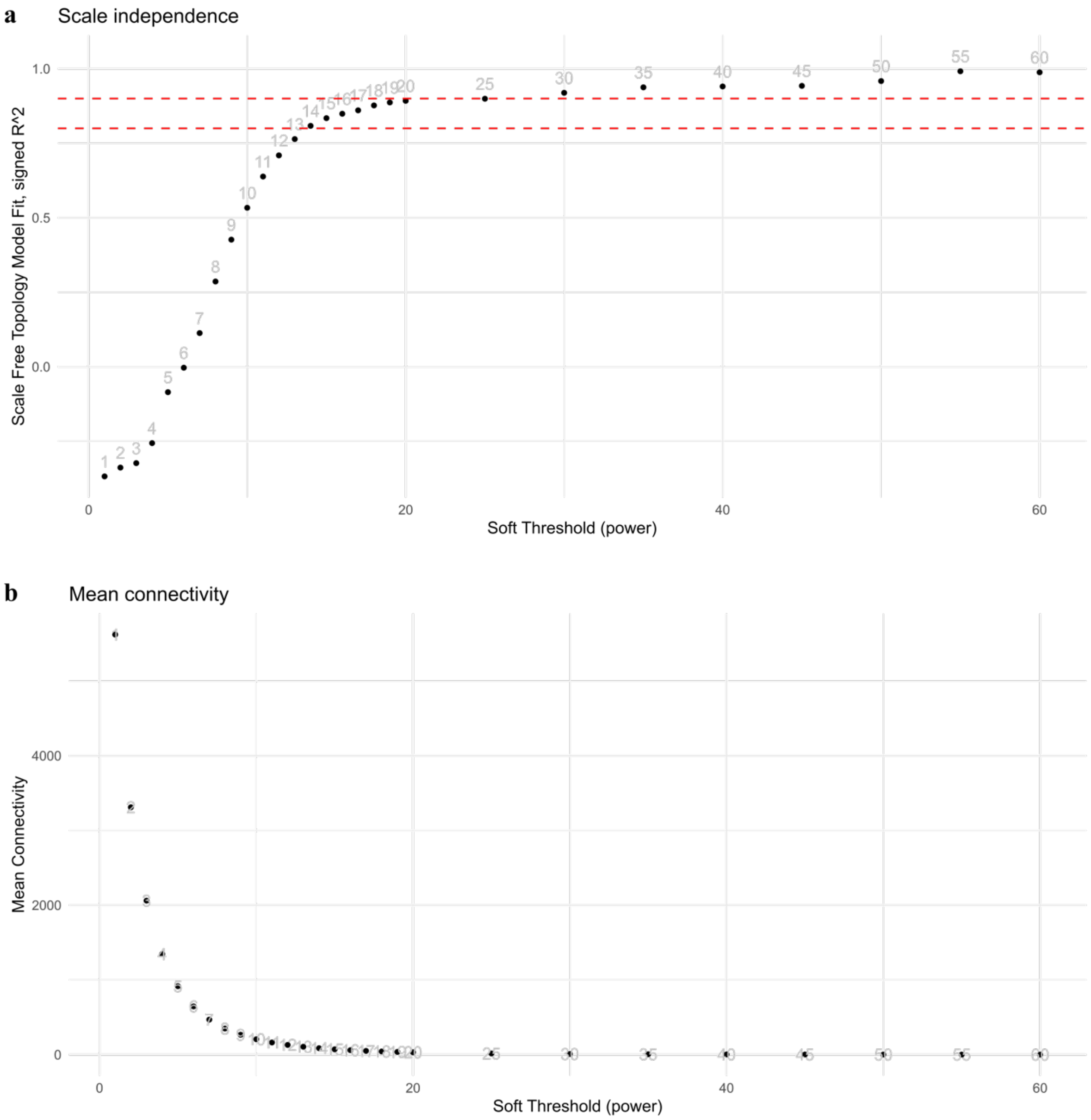

Supplementary Figure 22. Determination of soft-thresholding power and mean connectivity in WGCNA performed on the TPM expression values of KEGG KO identifiers. a) The scale-free topology model fit (signed R^2) as a function of soft thresholding powers. Each point represents the model fit index for a given soft threshold power, with grey

labels indicating specific power values. Red horizontal dashed lines at R^2 = 0.80 and R^2 = 0.90 indicate common thresholds for acceptable model fit. **b)** Mean connectivity as a function of the same soft thresholding powers, illustrating how the connectivity of the signed network changes with different thresholding powers.

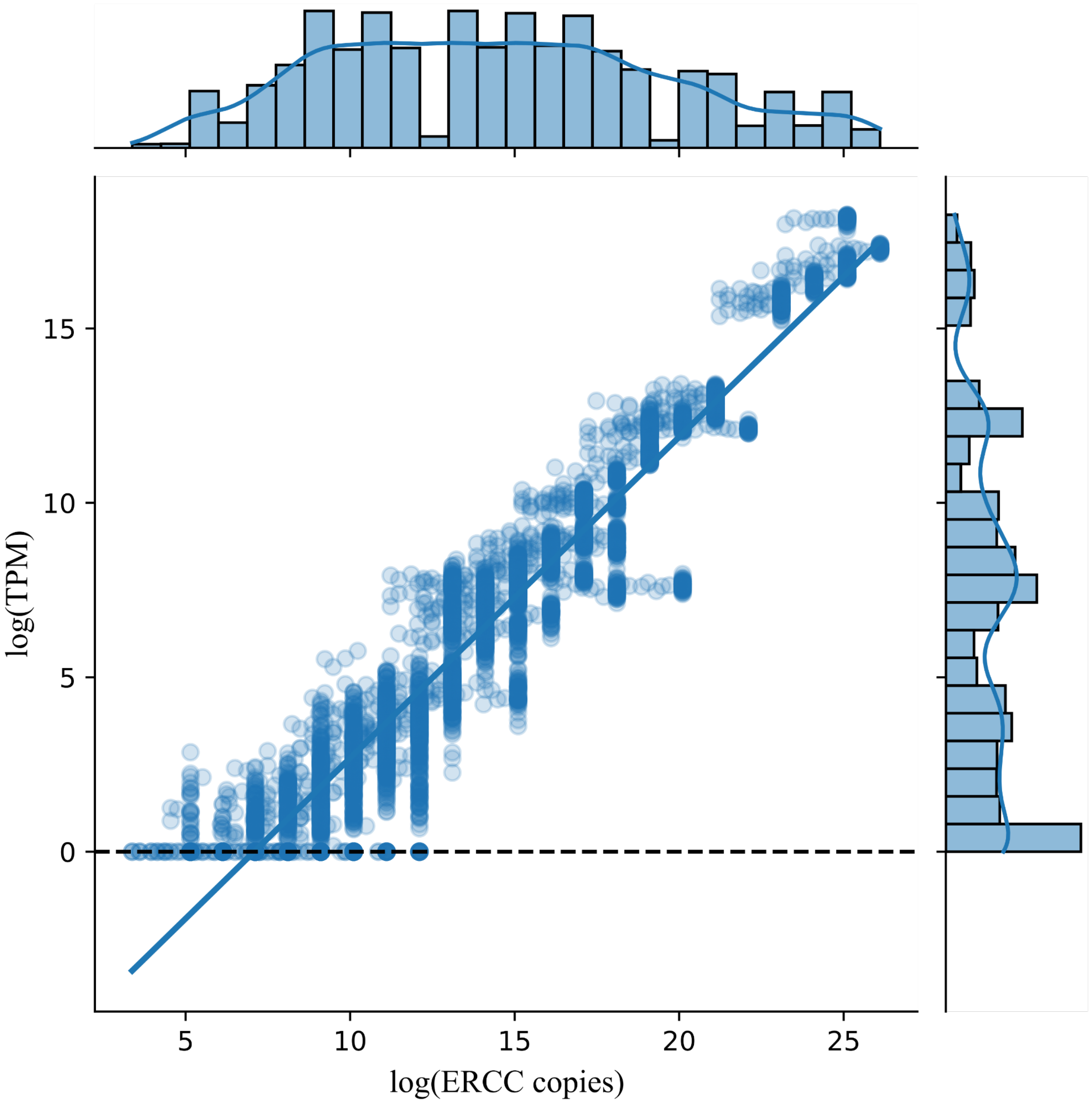

Supplementary Figure 23. The relationship between ERCC92 standards and their associated TPM counts. Jointplot illustrating the relationship between log2-transformed ERC992 transcript standards copies and their corresponding log-transformed transcript per million counts. The fitted trendline helps determine the lower limit of detection of expression quantification.

Supplementary Tables

	All stations			Nearshore			Offshore		
	Mean (± SD)	Min (month)	Max (month)	Mean (± SD)	Min (month)	Max (month)	Mean (± SD)	Min (month)	Max (month)
Temperature (°C)	12.34 (5.96)	2.17 (2021-02)	22.54 (2020-08)	12.46 (6.43)	2.17 (2021-02)	22.54 (2020-08)	12.22 (5.59)	3.02 (2021-02)	21.76 (2020-08)
Salinity (PSU)	32.99 (1.35)	29.92 (2021-02)	34.73 (2020-11)	32.42 (1.5)	29.92 (2021-02)	34.67 (2020-07)	33.54 (0.93)	31.74 (2021-05)	34.73 (2020-11)
Nitrate (µmol/L)	12.45 (13.68)	0.25 (2021-06)	57.82 (2021-02)	15.84 (16.94)	0.47 (2021-05)	57.82 (2021-02)	9.19 (8.74)	0.25 (2021-06)	29.32 (2021-01)
Nitrite (µmol/L)	0.43 (0.36)	0.03 (2021-02)	1.54 (2020-09)	0.54 (0.42)	0.03 (2021-07)	1.54 (2020-09)	0.32 (0.25)	0.03 (2021-02)	1.04 (2020-09)
Phosphate (µmol/L)	0.41 (0.39)	0 (2021-06)	1.4 (2021-02)	0.49 (0.46)	0 (2021-06)	1.4 (2021-02)	0.32 (0.3)	0 (2021-06)	0.96 (2021-01)
Silicate (µmol/L)	7.4 (9.29)	0 (2020-11)	43.86 (2021-02)	10.66 (11.51)	0 (2021-05)	43.86 (2021-02)	4.26 (4.96)	0 (2020-11)	16.26 (2021-01)
SPM (mg/L)	50.18 (58.07)	9 (2021-05)	340 (2021-01)	77.94 (73.63)	16.9 (2021-05)	340 (2021-01)	25.1 (16.46)	9 (2021-05)	75 (2021-02)

Supplementary Table 1 Summary of abiotic environmental variables measured in the Belgian Part of the North Sea. This table presents mean values of temperature, salinity, nitrate, nitrite, phosphate, silicate, and suspended particulate matter (SPM) data for all, nearshore (station 120, 130, and 700), and offshore (station ZG02, 330, and 780) stations. Additional environmental monitoring data can be found in Supplementary Dataset 1.

The North Sea micro-eukaryotic metatranscriptome

A total of 1.049 billion raw reads were generated from sea surface water samples, an average of 16 million (SD=2.3M) per sample. The resulting de novo metatranscriptome assembly contained over 7 million unique transcripts with a median transcript length of 342 bp (Fig. S1). 3,705,883 proteins were predicted from the assembled transcripts. Functional or taxonomic annotation information was found for 79% of predicted proteins. 2,235,576 proteins, 60% of the total, could be functionally annotated (Fig. S1c & d). Shallow taxonomic annotations, here defined as 60% sequence identity with either the PhyloDB or the EukProt reference databases, were found for 59% and 64% of proteins respectively. When using a stricter cut-off value of 90% sequence identity to obtain deeper taxonomic resolution, 26% of proteins could be assigned a taxonomic identity using PhyloDB and 13% matched with a EukProt reference sequence. Given the broader representation of eukaryotic diversity and more recent release date, we used the EukProt reference database for further analyses. However, for specific taxonomic groups, such as diatoms and dinoflagellates, both databases were consulted to obtain a more comprehensive picture. To further assess the representation of southern North Sea species in other global ocean reference databases, we examined the alignment of our assembled transcripts to the Tara Oceans' metagenome assembled genomes (MAGs) database[1] (retaining only alignments with at least 80% coverage and 95% sequence identity). On average, 2.59% of the assembled transcripts mapped to the collection of Tara oceans' eukaryotic genomes. For April samples, however, we observed higher mapping rates with 14% of the assembled transcripts mapping to the MAGs, due to high mapping rates against *Phaeocystis* genomes. The overall low taxonomic representation in the MAGs database indicates an underrepresentation of microeukaryotic plankton species from our study area in global ocean reference databases.

This highlights the importance of sampling, extracting, and characterising genomic data from the southern North Sea and similar temperate coastal marine ecosystems.

Supplementary Methods

Calculating cell densities from FlowCam image data

To obtain microphytoplankton biomass estimates, 50 L of sea surface water was collected using a stainless-steel bucket and filtered through a 55 µm mesh size Apstein net. Filtered samples were transferred to a plastic falcon and fixed with acidic Lugol's iodine solution (1-5 %). Samples were then stored in the dark at 4 °C until laboratory processing. In the lab, samples were processed using the FlowCam VS-4 bench-top model (Fluid Imaging Technologies, Yarmouth, Maine, U.S.A.) equipped with a Sony XCD SC90 digital grey-scale camera and VisualSpreadsheet® software (version 4.2.52). Samples were prefiltered at 300µm to avoid clogging of flowcells and a predefined threshold ensured only particles in the 50-300µm range were captured. A first presample run was used to determine particle load and determine sample dilution factors if Particles Per Used Image exceeded values of 1.2. Three replicate runs of each sample were carried out to minimise technical variation. The resulting output, consisting of collages of particle images, a set of image parameters, and sampling and processing metadata, were processed and uploaded to the internal BioSens MongoDB database. Unvalidated single images were then fed to a convolutional neural network, trained on an extensive dataset of human-validated images, that classified each particle into one of over 90 distinct taxonomic groups[2]. All model predictions were verified by human taxonomists.

Cell densities of the assigned taxonomic groups were calculated using the filtered volume of sea surface water, the volume of the sample measured in the lab, the fluid volume imaged by

FlowCam, the groups' particle count, and a dilution factor calculated from the dilution of samples:

$$group\ density\ (\frac{cells}{L}) = \frac{group\ particle\ count\ \times\ sample\ volume\ (mL)}{fluid\ volume\ imaged\ (mL)\ \times\ dilution\ factor\ \times\ volume\ filtered\ (L)}$$

The resulting cell densities were checked for possible outliers. The validated FlowCam data is made available through the Belgian LifeWatch RShiny application at https://rshiny.vsc.lifewatch.be/flowcam-data/. For more detailed information on the full FlowCam protocol see Martínez et al. (2020)[2].

Transcript and ERCC spike quantification & normalisation

Transcript and ERCC spike-in abundances were quantified using Kallisto, yielding TPM (Transcript Per Million) counts for every transcript[3]. Using the known molar concentrations of the 92 RNA spikes, a lower limit of detection (LLD) could be calculated (Fig. S21). The LLD is the concentration of a given transcript (attomoles/µL), or the number of copies, that needs to be present in a sample to yield one TPM count. TPM counts below the trusted LLD were set to 0. We calculated transcripts per L using ERCC RNA spike-in recovery values according to:

$$\begin{aligned} transcripts_{i,j}L^{-1} \\ &= TPM_{i,j} \times \frac{spike\ copies_j}{spike\ TPM_j} \times \frac{resuspension\ volume_j}{volume\ used\ for\ RNA\ extraction_j} \\ &\times \frac{total\ extracted\ RNA_j}{RNA\ used\ for\ LP_j} \times \frac{1}{volume\ filtered_j} \end{aligned}$$

Where $transcripts_{i,j}L^{-1}$ equals the amount of RNA copies per L of seawater of transcript *i* in sample *j*, $TPM_{i,j}$ are the TPM values of transcript *i* in sample *j*, $spike\ copies_j$ is the average amount of spike copies added to sample *j*, spike TPM_j are the average amount of spike TPM in

sample *j*, *volume filtered*$_j$ is the volume of seawater filtered for sample *j* (in L), *resuspension volume*$_j$ is the amount of seawater used to re-elute the filtration residue, *RNA used for LP*$_j$ is the amount of RNA used for sequencing library preparation of sample j, and *total extracted RNA*$_j$ is the amount of total RNA extracted from sample j[4]. In calculating the amount of RNA copies per L of seawater we ignored the loss of extracted RNA for quality control, as these volumes were equal for all samples.

References

1. Delmont, T. O. *et al.* Functional repertoire convergence of distantly related eukaryotic plankton lineages abundant in the sunlit ocean. *Cell Genomics* **2**, 100123–100123 (2022).
2. Martínez, L. A., Mortelmans, J., Dillen, N., Debusschere, E. & Deneudt, K. LifeWatch observatory data: phytoplankton observations in the Belgian Part of the North Sea. *Biodivers. Data J.* **8**, (2020).
3. Bray, N., Pimentel, H., Melsted, P. & Pachter, L. Near-optimal probabilistic RNA-seq quantification. *Nat. Biotechnol.* **34**, 525–527 (2016).
4. Natalie R. Cohen, Harriet Alexander, Arianna I. Krinos, Sarah K. Hu, & Robert H. Lampe. Marine Microeukaryote Metatranscriptomics: Sample Processing and Bioinformatic Workflow Recommendations for Ecological Applications. *Front. Mar. Sci.* (2022) doi:10.3389/fmars.2022.867007.
